# Tagging of *C. elegans* apoptosis activator EGL-1 BH3-only reveals CED-9 BCL-2-dependent mitochondrial localization and dynamic control of EGL-1 synthesis and degradation in vivo

**DOI:** 10.1038/s41418-026-01682-0

**Published:** 2026-02-11

**Authors:** Yanwen Jiang, Kate JE Hodgson, Ioannis Segos, Eric J. Lambie, Lumeng Yang, Minjia Pan, Alan Greig, Barbara Conradt

**Affiliations:** 1 Cell and Developmental Biology, The Centre for Cell and Molecular Dynamics, Faculty of Life Sciences, University College London, London, UK; 2 Present Address: CHUM Research Center (CRCHUM), University of Montreal, Montreal, QC Canada; 3 Present Address: Helmholtz Zentrum Munich, Neuherberg, Germany; 4 Present Address: Department of Physiology, Development and Neuroscience, University of Cambridge, Cambridge, UK

**Keywords:** Proteolysis, Development, Molecular biology

## Abstract

The BH3-only protein EGL-1 is the key activator of apoptosis during *C. elegans* development. EGL-1 protein is thought to be synthesized predominantly in cells programmed to die and to localize to mitochondria. We used CRISPR-Cas-mediated modification of the *egl-1* locus to add the coding sequence for the monomeric StayGold fluorescent protein or 18 copies of the SunTag peptide to the endogenous open reading frame. We found that tagged EGL-1 protein colocalizes with mitochondria in vivo and that mitochondrial localization is dependent on the anti-apoptotic BCL-2-like protein CED-9. Consistent with the presence of *egl-1* mRNA in cells programmed to die as well as their progenitor cells (‘mother’ cells), EGL-1 protein is detected in both types of cells in vivo. Furthermore, real time imaging reveals that EGL-1 protein rapidly disappears from the mother cell prior to its division and that EGL-1 protein rapidly reappears specifically in the daughter cell programmed to die. Our results demonstrate CED-9 BCL-2-dependent mitochondrial localization of EGL-1 BH3-only protein and dynamic control of EGL-1 protein synthesis and degradation. Furthermore, we have identified additional levels of control of *egl-1* BH3-only function that expand our understanding of apoptosis activation in vivo.

## Introduction

Apoptosis is the predominant type of programmed cell death during animal development, and it is used to sculpt the organism and eliminate ‘unwanted’ cells [1–5]. BH3-only proteins, pro-apoptotic members of the BCL-2 superfamily of apoptosis regulators, are key activators of apoptosis in animals as diverse as nematodes and mammals [6, 7]. ABT-199 (also known as Venclexta or Venetoclax), a small compound that mimics the pro-apoptotic function of BH3-only proteins and inactivates the anti-apoptotic protein BCL-2, was approved for the treatment of chronic lymphocytic leukemia (CLL) in 2016 and has since successfully been used in the clinic [8–10]. This underscores the pivotal roles of apoptosis and BH3-only proteins in both normal development and tumorigenesis.

Through a series of genetic studies using *C. elegans* as a model, genes encoding core components of the apoptosis pathway were identified and their sequential functions determined [11–13]. Subsequent molecular characterization of the corresponding gene products revealed that the pathway comprises EGL-1 BH3-only, CED-9 BCL-2, CED-4 APAF-1, and CED-3 Caspase [14–17]. At least during embryonic development, the *ced-9*, *ced-4* and *ced-3* genes are expressed ubiquitously, and their gene products are present in all cells, associated with mitochondria (a fraction of CED-3 is also found in the cytoplasm) [18, 19]. There are two pools of CED-4 protein: one pool is found all along the length of mitochondria, and the localization to mitochondria of this pool is dependent on CED-9; a second pool is found in foci that are closely associated with mitochondria, and the association with mitochondria of this pool is independent of CED-9 [19]. The gene *egl-1* is required and sufficient for apoptosis and acts upstream of *ced-9*, *ced-4*, and *ced-3* [14]. Over-expression of *egl-1* in embryos causes re-localization of the pool of CED-4 protein found along mitochondria into mitochondria-associated CED-4 foci. This may constitute a critical step in apoptosome assembly and CED-3 Caspase activation and, hence, apoptosis activation [19].

Expression of *egl-1* is largely restricted to lineages in which a programmed cell death occurs (‘cell death’ lineages), and this is mediated by a combination of control at the epigenetic, transcriptional, and post-transcriptional levels [20–22]. *egl-1* mRNA is found at low concentrations in mothers of cells programmed to die and at higher concentrations in daughters programmed to die [21–23]. However, the presence of EGL-1 protein in these two types of cells has not been demonstrated. Furthermore, EGL-1 protein can bind to CED-9 BCL-2 in vitro, in yeast and in mammalian cells grown in culture, and this has led to the prediction that EGL-1 protein localizes to mitochondria [5, 14, 24, 25]. However, CED-9-dependent localization of EGL-1 to mitochondria has not been demonstrated in vivo.

To begin to address these questions, we sought to develop genetic tools that would permit in vivo visualization of endogenous EGL-1 BH3-only protein during *C. elegans* development. To this end, we used CRISPR-Cas mediated editing of the *egl-1* locus to generate monomeric StayGold- and SunTag-tagged versions of endogenous EGL-1 protein.

## Results

### Tagging *egl-1* BH3-only with monomeric StayGold (*dx243* allele)

**Fig. 1:**
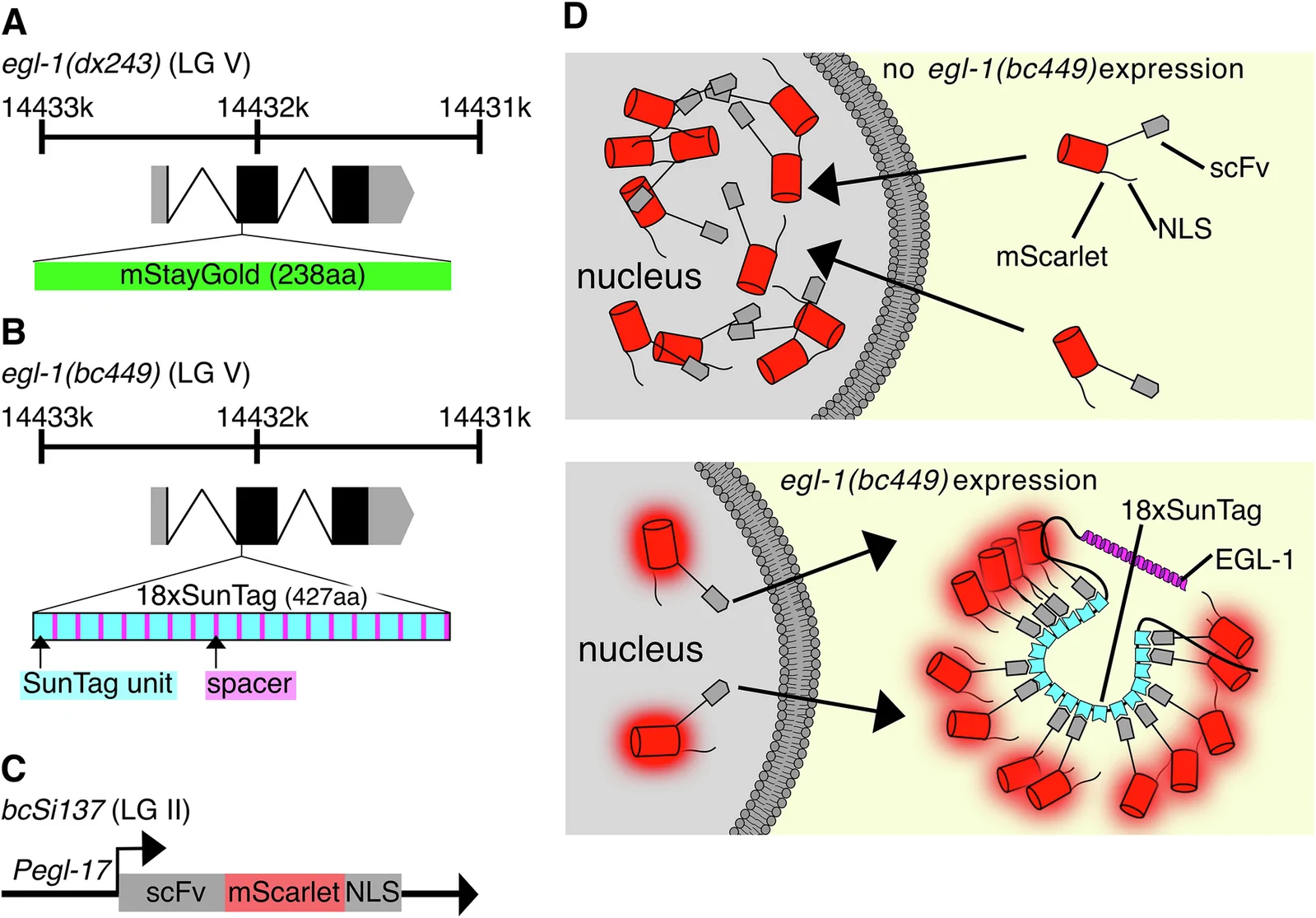
Schematics of *egl-1*(*dx243*) and *egl-1*(*bc449*) alleles and SunTag-based strategy for visualizing EGL-1 protein. **A**, **B** mSG and 18xSunTag insertion sites in *egl-1* locus on linkage group (LG) V. Gene structures drawn according to annotations on WormBase [51, 52]. **C** Schematic of transgene *egl-17*p::scFv::mScarlet::GB1::NLS::*pie-1* 3′ UTR (pBC2020) used to generate *bcSi137*. **D** SunTag-based strategy for visualizing EGL-1 protein.

We used CRISPR-Cas12 editing to modify the genome of wild-type N2 worms (see “Methods”), and candidate insertion alleles were sequenced to validate the structures of the edited loci. The strategy for tagging the *egl-1* gene is diagrammed in Fig. 1A. We used publicly available data [14] (wormbase.org) to choose a tag location that would be unlikely to interfere with protein activity, proper localization, or interaction with CED-9. We used monomeric StayGold (mSG) for tagging (*dx243* allele), because it is expected to be superior to GFP and mNeonGreen in terms of brightness and resistance to photobleaching [26].

**Fig. 2:**
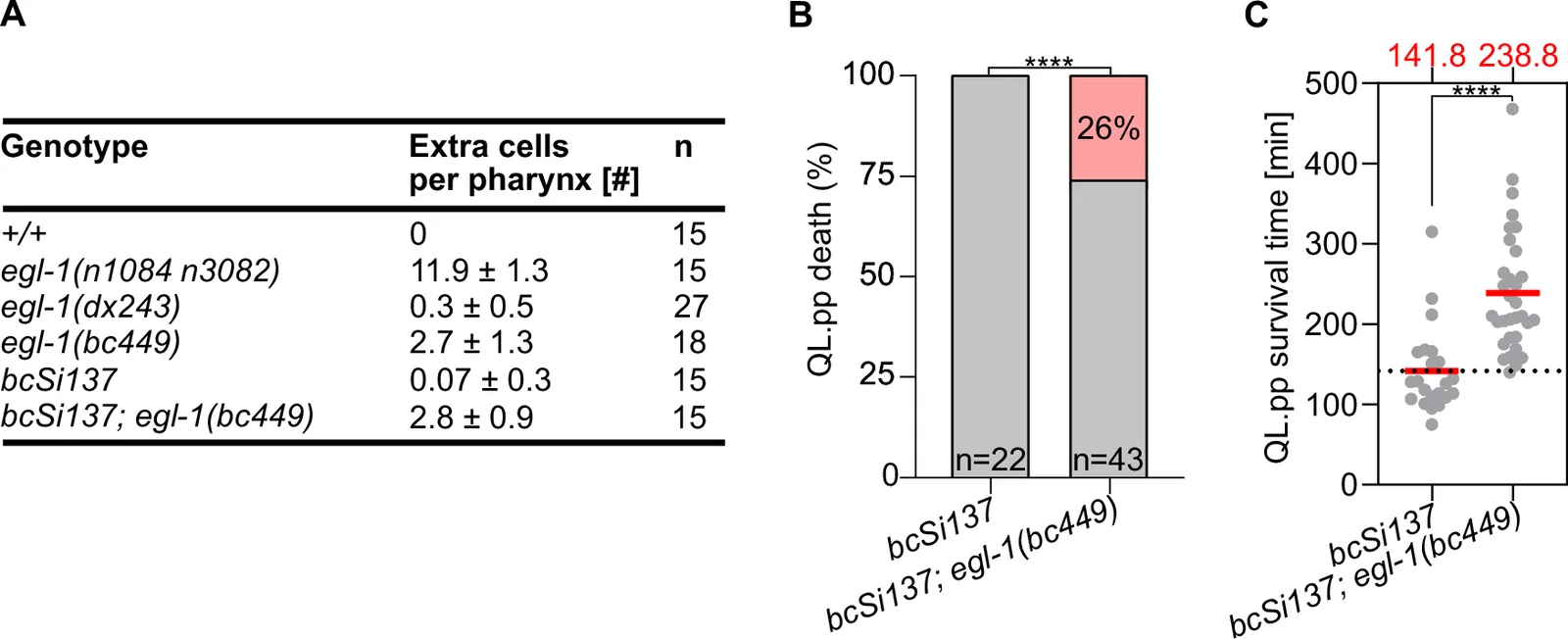
Cell death defects in *egl-1*(*dx243*) and *egl-1*(*bc449*) animals. **A** Average number of extra cells ± standard deviation in the anterior pharynx in animals of various genotypes. Extra cells were determined as described in “Methods”. n, number of pharynxes scored. **B** Inappropriate QL.pp survival and **C** QL.pp survival time as described in “Methods”. *n* = 22 and *n* = 43 for *bcSi137* (*+/+*) and *bcSi137; egl-1(bc449)*, respectively. (Both strains also contained the transgene *bcIs158*.) P values are calculated using the Fischer’s exact test (**B**), and the Mann–Whitney test (**C**). Normality was tested with the Shapiro-Wilk test. * = *P* value ≤ 0.05; * =: *P* value ≤ 0.01; *** = *P* value ≤ 0.001; **** = *P* value ≤ 0.0001. Red bar and values in (**C**) indicate the population average.

We found that the homozygous tagged strain is viable and appears healthy. In order to quantitatively assess the impact of mSG-tagged EGL-1 (mSG::EGL-1) on apoptosis during embryonic development, we counted the number of ‘extra’ cells in the anterior pharynx of L4 stage hermaphrodites [27]. Strong loss of function alleles of *egl-1* such as *n1084 n3082* cause the presence of on average 11.9 extra cells within this region [14] (Fig. 2A). For comparison, in wild-type animals (+/+), 0 extra cells are present. We found that the mSG-tagged allele *dx243* causes on average 0.3 extra cells, suggesting that there is no major alteration in the function of the *egl-1* gene when the 238 amino acid mSG protein is fused to the endogenous 106 amino acid EGL-1 protein (wormbase.org).

### Presence of mSG::EGL-1 protein in embryos assayed by widefield microscopy

**Fig. 3:**
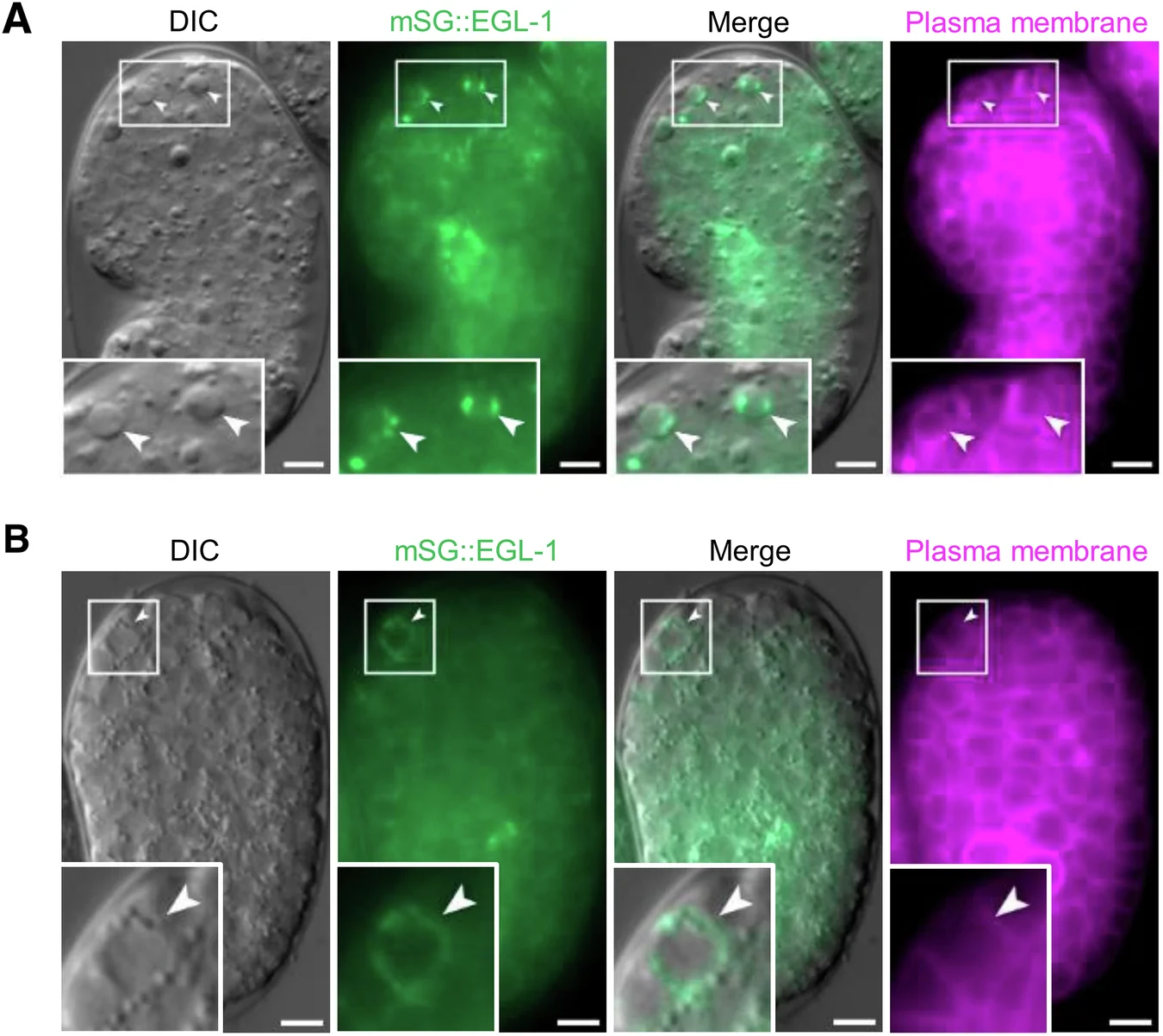
Expression and localization of mSG::EGL-1 in live embryos. **A** mSG::EGL-1 expression and localization in cells programmed to die (cell corpses). White arrows mark the location of refractile apoptotic cells, identified using Differential Interference Contrast (DIC) microscopy. **B** mSG::EGL-1 expression and localization in a non-apoptotic cell, presumed to be the mother of a cell programmed to die. The white arrow indicates the non-apoptotic cell containing mSG::EGL-1 signal. All images were acquired on a Zeiss Axio Imager M2 microscope as described in “Methods”. Representative examples are shown. Genotype of animals analysed *ltIs44 egl-1(dx243)* V. mSG::EGL-1 (*dx243*, green) panels in (**A**) and (**B**) were set to the same brightness scale in ImageJ. Plasma membranes were labeled with *pie-1p::mCherry::PH*(*PLC1δ1*) (*ltIs44*, magenta). Images were colorised, composited, and annotated in ImageJ, converted to 8-bit, and compiled in Microsoft PowerPoint^TM^. Scale bar, 2 µm.

Each of the CED-9 BCL-2, CED-4 APAF-1, and CED-3 Caspase proteins is present in most if not all cells of the embryo [18, 19]. In contrast, *egl-1* expression is tightly controlled and *egl-1* mRNA found essentially only in cell death lineages [20–23]. Specifically, *egl-1* mRNA is found at low concentrations in mothers of cells programmed to die and at higher concentrations in cells programmed to die. Consistent with the spatiotemporally restricted pattern of *egl-1* expression, using widefield microscopy (Zeiss Axio Imager 2), mSG::EGL-1 is present in only a few cells of a live embryo (Fig. 3). (For autofluorescence emission in embryos of that stage, see Fig. S1) Using Differential Interference Contrast (DIC) microscopy, apoptotic cells can be recognized by their refractile button-like appearance [28, 29]. Based on DIC, we found that about half of mSG::EGL-1 positive cells are apoptotic cells (Fig. 3A; Fig. S2). Furthermore, surveying apoptotic cells for the presence of mSG::EGL-1, we found that almost two-thirds have detectable levels of mSG::EGL-1 (64%; *n* = 39). Based on the presence of low concentrations of *egl-1* mRNA also in mothers of cells programmed to die, the non-refractile mSG::EGL-1 positive cells most likely represent mother cells (Fig. 3B; Fig. S3). Therefore, unlike CED-9 BCL-2, CED-4 APAF-1, and CED-3 Caspase proteins, EGL-1 BH3-only protein is found only in a few cells of the embryo. These cells include cells programmed to die and most likely mothers of cells programmed to die.

### Subcellular localization of mSG::EGL-1 protein assayed by super-resolution imaging

**Fig. 4:**
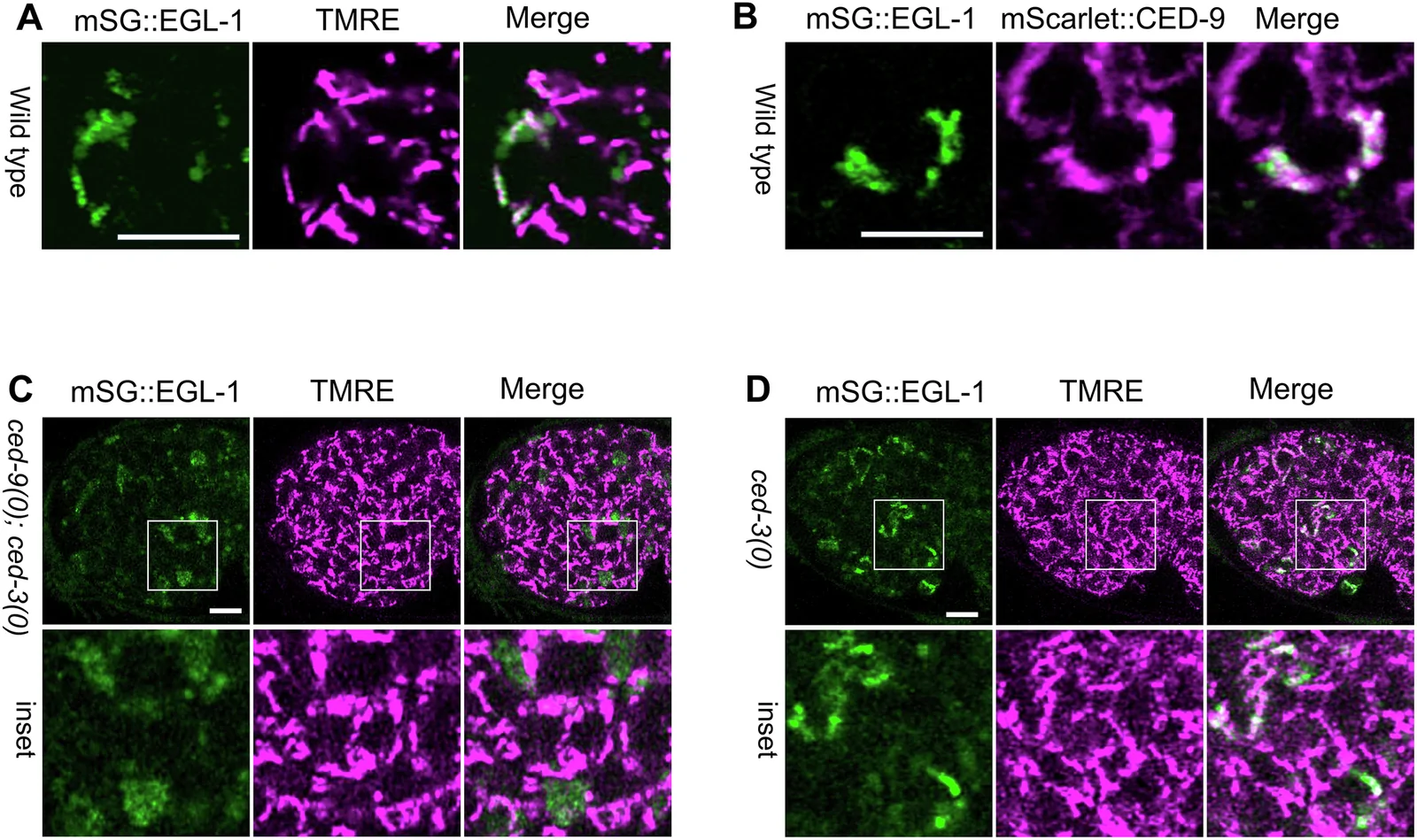
Subcellular localization of mSG::EGL-1 in live embryos. **A** Mitochondrial localization of mSG::EGL-1. Embryos of genotype *egl-1(dx243)* were stained with TMRE to label mitochondria (“Methods”). **B** Colocalization of mSG::EGL-1 and mScarlet::CED-9. Genotype *ced-9(n6731)* III; *egl-1(dx243)* V. **C** Mitochondrial localization of mSG::EGL-1 is dependent on CED-9. Genotype *ced-9(dx212); ced-3(dx213)* IV; *egl-1(dx243)* V. **D** Mitochondrial localization of mSG::EGL-1 is not dependent on CED-3. Genotype *ced-3(dx213)* IV; *egl-1(dx243)* V. Images were acquired using a Zeiss LSM 980 equipped with an Airyscan detector, as described in “Methods”. Representative examples are shown.

Particularly in non-refractile cells, mSG::EGL-1 labels tubular subcellular structures that resemble mitochondria (Fig. 3B; Fig. S3). To test whether the subcellular localization of mSG::EGL-1 overlaps with mitochondria, we stained live embryos with TMRE (Tetramethylrhodamine ethyl ester) [30], which is selectively concentrated in the mitochondrial matrix due to the inner mitochondrial membrane (IMM) potential. Consistent with the proposed localization of EGL-1 to mitochondria [5, 14, 24, 25], using a Zeiss LSM 980 microscope equipped with an Airyscan 2 detector (“Methods”), we observed identical patterns in both channels i.e., for the mStayGold and TMRE signal (Fig. 4A).

CED-9 BCL-2 localizes to the outer mitochondrial membrane (OMM) in live embryos [19] and interacts with EGL-1 in vitro, in yeast and in mammalian cells grown in culture [14, 25]. To confirm the interaction of EGL-1 with CED-9 in vivo, we crossed the mSG::EGL-1 allele *dx243* into a strain homozygous for the *ced-9* allele *n6731*, which generates functional mScarlet-tagged CED-9 protein (mScarlet::CED-9) [31]. Consistent with the proposed interaction between CED-9 and EGL-1, we observed colocalization of the mScarlet and mStayGold signal (Fig. 4B).

To test whether EGL-1 localization to mitochondria is dependent on CED-9, we analyzed mSG::EGL-1 subcellular localization in *ced-9*(0); *ced-3*(0) animals stained with TMRE. (To rescue the embryonic lethal phenotype of *ced-9*(0), this experiment was performed in the background of *ced-3*(0).) When comparing mSG::EGL-1 and TMRE, we found that the identical patterns observed in both channels in a wild-type genetic background are essentially lost in *ced-9*(0); *ced-3*(0) embryos (Fig. 4C). Instead, mSG::EGL-1 adopts a predominantly cytoplasmic localization. The shift in localization pattern of mSG::EGL-1 is not due to the *ced-3*(0) mutation, because a strain of genotype *ced-3*(0); *egl-1*(*dx243*) has the same distribution of mSG::EGL-1 as in wild type (Fig. 4D). Together, these results demonstrate that in vivo, EGL-1 BH3-only localizes to mitochondria in a CED-9 BCL-2-dependent manner.

### Presence of EGL-1 protein in the embryonic NSM neuroblast lineage

**Fig. 5:**
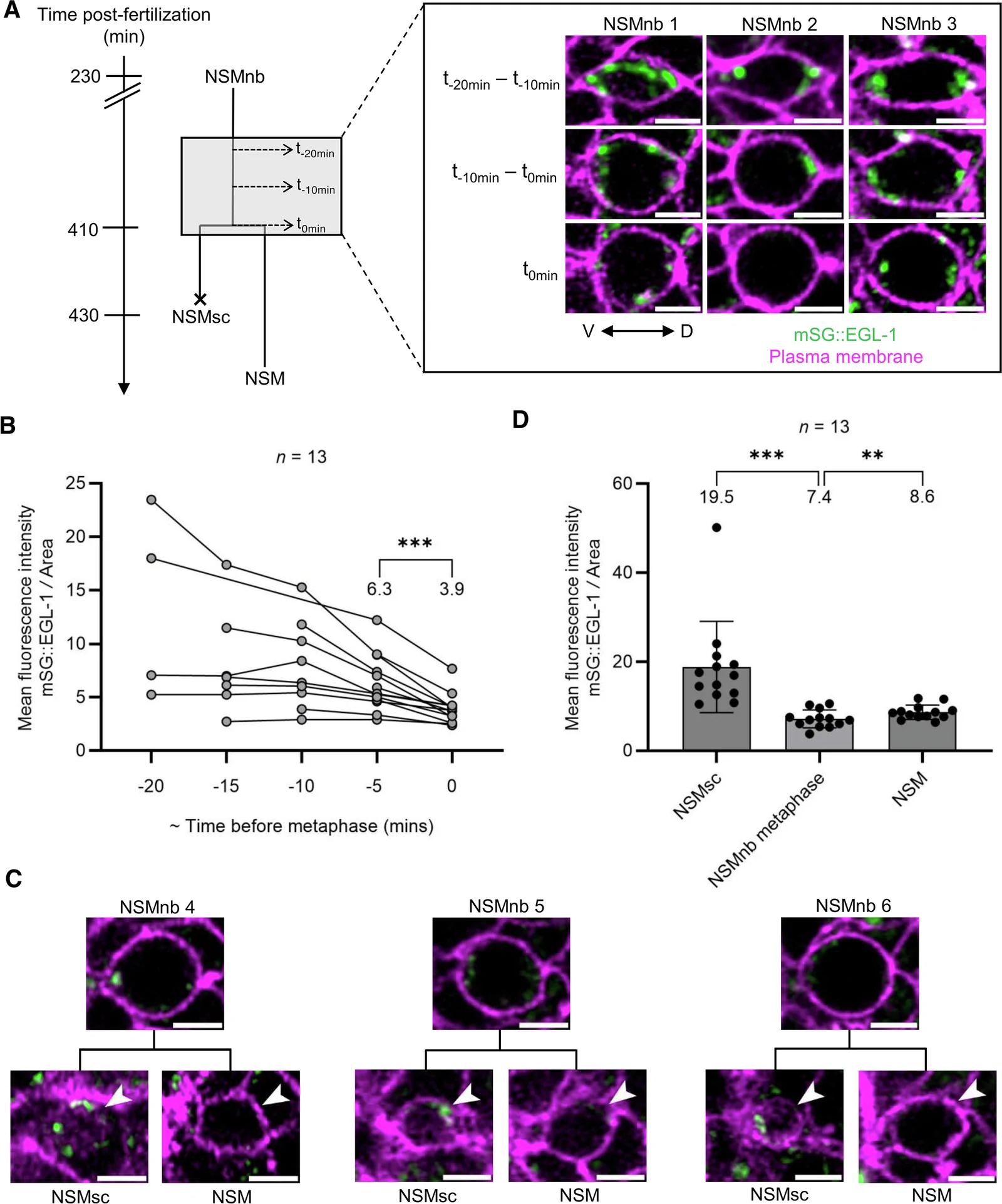
Dynamics of mSG::EGL-1 in the embryonic NSMnb lineage in live embryos. **A** Schematic of the embryonic NSM lineage. The gray box indicates the imaging window (t_−20 min_ to t_0min_, with t_0min_ marking metaphase). NSM neuroblasts (NSMnb) were identified in late randomly selected comma-stage embryos by their distinct position and morphology using the plasma-membrane marker *pie-1p::mCherry::PH*(*PLC1δ1*) (*ltIs44*, magenta). Central Z-slices of representative wild-type NSMnbs expressing mSG::EGL-1 (*dx243*, cyan) and *pie-1p::mCherry::PH*(*PLC1δ1*) (*ltIs44*, magenta) are shown in the right box projected from the imaging window. D, dorsal; V, ventral. Scale bar, 2 μm. Three representative examples are shown. **B** Quantification of mSG::EGL-1 signal intensity per NSMnb (total fluorescence divided by cell area) over time up to metaphase (t_0min_) (*n* = 13). Gray circles connected by black lines represent measurements from the same NSMnb. Time points are approximate ( ± 2 min). Statistical comparison between time points t_-5 min_ and t_0min_ was performed using the Wilcoxon signed-rank test (*** = *P* < 0.001). **C** Central Z-slices of representative wild-type NSMnbs at metaphase and of their daughter cells (NSMsc and NSM), expressing mSG::EGL-1 (*dx243*, cyan) and *pie-1p::mCherry::PH*(*PLC1δ1*) (*ltIs44*, magenta). Images connected by black lines derive from the same lineage. White arrows indicate the locations of the NSMsc and NSM within the image frame. Scale bar, 2 μm. **D** Quantification of mSG::EGL-1 signal intensity per cell (total fluorescence divided by cell area) in NSMnbs at metaphase and in their daughter cells (NSMsc and NSM), measured 20–30 min after NSMnb metaphase (*n* = 13). Data points for NSMsc and NSM are paired with their corresponding NSMnb metaphase measurement to allow direct comparison. Mean values are indicated above each bar and ±SD values are overlayed on each bar. Statistical significance was determined by the Wilcoxon signed-rank test (** = *P* < 0.01, *** *P* = < 0.001). Genotype of animals analysed *ltIs44 egl-1(dx243)* V. Images were acquired on a Zeiss LSM 980 microscope equipped with an Airyscan 2 detector, as described in the “Methods”. GraphPad Prism™ was used for statistical analyses and for generating the graphs shown.

Next, we analyzed mSG::EGL-1 at super resolution and in real time in a specific embryonic cell death lineage, the NSM neuroblast (NSMnb) lineage [29]. To that end, we used a Zeiss LSM 980 microscope equipped with an Airyscan 2 detector (“Methods”). The NSMnb divides asymmetrically and generates a larger daughter that differentiates into a serotonergic neuron, the ‘neurosecretory motorneuron’ (NSM), and a smaller daughter that is programmed to die, the NSM sister cell (NSMsc) [29] (Fig. 5A). Using a plasma membrane-localized mCherry reporter (*ltIs44*), we can identify the NSMnb in live 1 ½-fold embryos based on its position and shape (“Methods”), observe its division ~410 min post-fertilization, and follow the fates of its daughter cells [32].

In all NSMnb analyzed (*n* = 13), we detected mSG::EGL-1 before they rounded up and entered metaphase (Fig. 5A). Also, in all NSMnb analyzed (*n* = 13), the mSG::EGL-1 signal subsequently rapidly disappeared. As a result, at the time the cells entered metaphase and divided (t_0min_), little or no mSG::EGL-1 remained (Fig. 5A–C; Fig. S4). Within ~22 min post-NSMnb division, the NSMsc dies and adopts a refractile button-like appearance [33]. We followed the NSMsc and its surviving sister (NSM) for up to 30 min post-NSMnb division and found that mSG::EGL-1 signal reappeared in all NSMsc analyzed (n = 13) (Fig. 5C, D; Fig. S4). In contrast, over the same time period, mSG::EGL-1 signal reappeared in none of the NSM (*n* = 13) (Fig. 5C, D; Fig. S4). Finally, we measured mean fluorescence intensities of mSG::EGL-1 signal per area and found that the intensity in the NSMsc was more than 2-fold higher than in the NSM or the NSMnb (at metaphase) (Fig. 5D).

As stated above, surveying live embryos for the presence of mSG::EGL-1, we found that one-third of apoptotic cells had no detectable levels of mSG::EGL-1. Based on the finding that it takes up to 30 min post-NSMnb division for mSG::EGL-1 to become detectable in the NSMsc, it is highly likely that in this one-third of apoptotic cells, at the time the embryos were surveyed, mSG::EGL-1 levels were not yet high enough to be detected.

In summary, our analysis of the embryonic NSMnb lineage demonstrates that EGL-1 protein is present in the cell programmed to die and its mother cell but not in the surviving sister cell. It also reveals that the presence of EGL-1 protein in cell death lineages is highly dynamic, with a rapid disappearance of EGL-1 protein prior to mother cell division and its rapid reappearance post-cytokinesis.

### Presence of EGL-1 protein in the post-embryonic QL.p neuroblast lineage

**Fig. 6:**
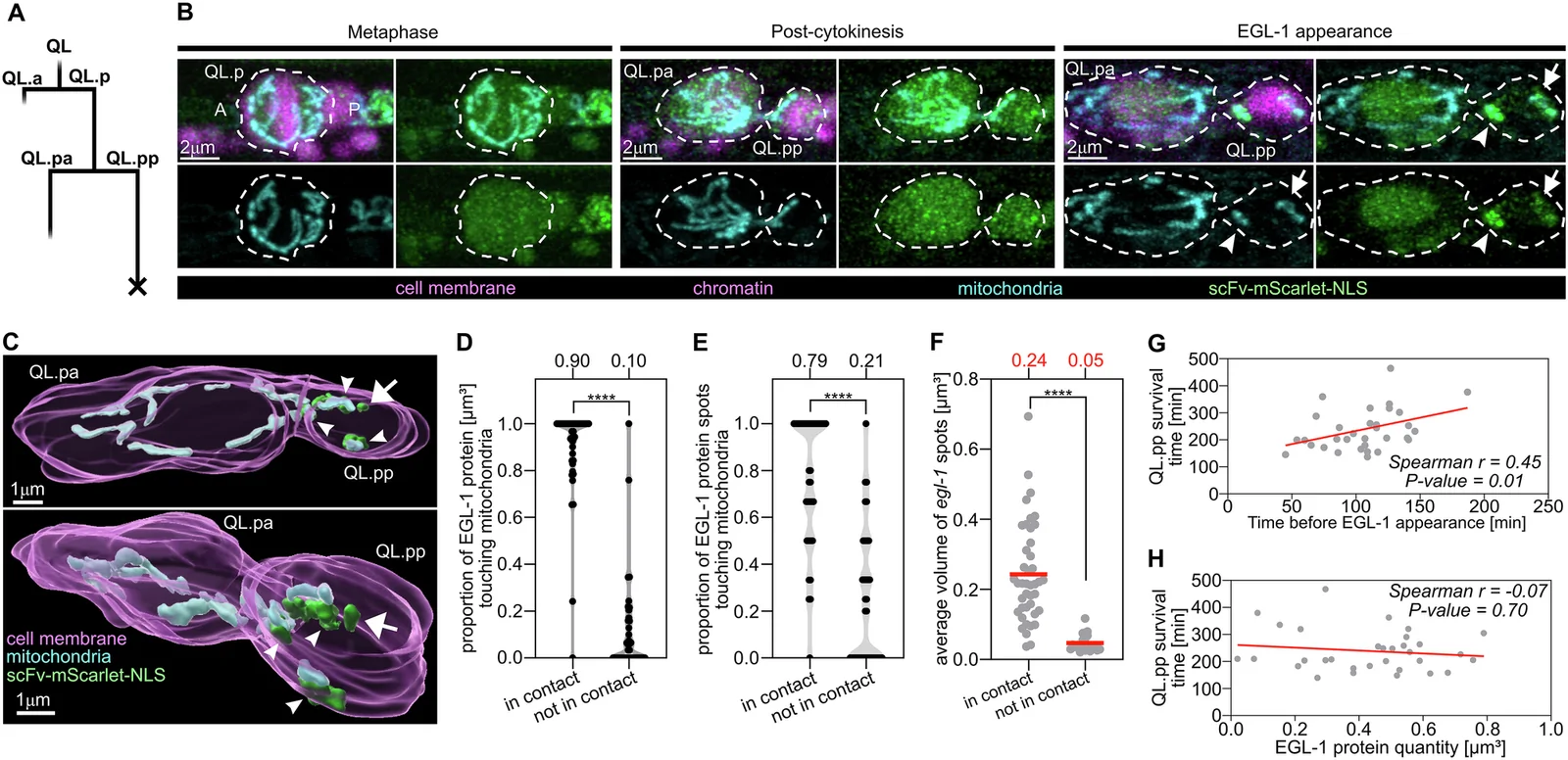
Subcellular localization and dynamics of 18xSunTag::EGL-1 protein in the post-embryonic QL.p lineage in live L1 larvae. **A** Schematics of the QL.p lineage. **B** Airyscan Super Resolution images of QL.p at ‘Metaphase’ (left), ‘Post-cytokinesis’ (center), and ‘EGL-1 appearance’ (right). All three time points refer to the same time point in Fig. 2. For each time point, three-channel (cell lineage markers (magenta), mitochondria (cyan), and scFv-mScarlet-NLS (green)) merged (top-left); two-channel (mitochondria (cyan) and scFv-mScarlet-NLS (green)) (top-right), mitochondria in cyan (bottom-left), and scFv-mScarlet-NLS (green) (bottom-right), are shown. **C** 3D rendering of cell membrane, mitochondria, and scFv-mScarlet-NLS recruited to 18xSunTag::EGL-1 (‘EGL-1 appearance’ time point). White arrowheads and arrows indicate EGL-1 clusters contacting or not contacting mitochondria, respectively. (This 3D example does not refer to the example illustrated in Part B). **D**, **E** Proportion of EGL-1 protein or protein spots touching (‘in contact’) mitochondria in QL.pp (*n* = 43 in box panels). **F** Average volume of EGL-1 spots for both ‘in contact’ and ‘not in contact’ EGL-1 spots. **G** Correlation between QL.pp time before EGL-1 appearance (x axes) and QL.pp survival time (y axes) (*n* = 32). **H** Correlation between EGL-1 protein quantity (x axes) and QL.pp survival time (y axes) (*n* = 32). *P* values are calculated using the Fischer’s exact test (**D**, **E**) and the Mann–Whitney test (**F**). Normality was tested with the Shapiro-Wilk test. * = *P* value ≤ 0.05; ** = *P* value ≤ 0.01; *** = *P* value ≤ 0.001; **** = *P* value ≤ 0.0001. In Part **F**, red lines represent the population average. All data are from animals of the genotype *bcSi137 II*; *egl-1(bc449) V; bcIs158*.

Next, we analyzed mSG::EGL-1 in a post-embryonic cell death lineage, the QL.p neuroblast lineage [28]. QLp divides asymmetrically in 1^st^ larval stage (L1) larvae and generates a larger daughter that survives (and divides to generate two neurons), QL.pa, and a smaller daughter that dies, QL.pp (Fig. 6A, B). Using methodology that we recently developed [34, 35], *egl-1*(*dx243*) L1 larvae were cultured and immobilized using polystyrene nanospheres and the QL blast cell identified using a Q lineage-specific reporter (*rdvIs1*) [36]. Using an inverted Zeiss LSM 980 microscope equipped with a GaAsP detector, we surveyed (in Airyscan super resolution mode) a large number of QL.p at different time points between their birth ~2 h 50 min after hatching and the completion of their division ~5 h after hatching (*n* = 111); however, we were unable to detect mSG::EGL-1 in any QL.p.

Therefore, we decided to use the SUperNova (Sun) Tag system [37–41] to amplify the fluorescence intensity signal obtained from each molecule of EGL-1 protein. In brief, the SunTag system is based on the interaction between a 19 amino acid peptide of the *S. cerevisiae* transcription factor GCN4 (referred to as ‘SunTag’) and a single-chain fragment variable (scFv) antibody against the SunTag peptide fused to an effector protein, such as a fluorescent protein. Using CRISPR-Cas12 mediated genome editing, we tagged *egl-1* with 18 SunTag copies (*bc449* allele; generating 18xSunTag::EGL-1 protein) (Fig. 1B). In addition, we generated an integrated single-copy transgene that mediates the Q lineage-specific synthesis of the SunTag scFv antibody fused to monomeric Scarlet (mScarlet) and a nuclear localization signal (NLS) (scFv::mScarlet::NLS) (*bcSi137* transgene, *egl-17*p::*scFv::mScarlet::NLS*) (Fig. 1C). In cells in which the *egl-1*(*bc449*) gene is not expressed and, hence, 18xSunTag::EGL-1 protein not synthesized, scFv::mScarlet::NLS protein is found in the nucleus (Fig. 1D, top). In cells in which *egl-1*(*bc449*) is expressed and 18xSunTag::EGL-1 protein is synthesized, scFv::mScarlet::NLS protein binds to the SunTag peptides of 18xSunTag::EGL-1 as the protein is being translated. Consequently, a fraction of scFv::mScarlet::NLS protein accumulates in the cytoplasm, where it decorates 18xSunTag::EGL-1 protein, thereby amplifying ‘EGL-1 signal’ and revealing its subcellular localization (Fig. 1D, bottom).

To test whether the co-expression of 18xSunTag::EGL-1 and scFv::mScarlet::NLS compromises *egl-1* function, we counted the number of extra cells in the anterior pharynx. We found that *egl-1*(*bc449*) and *bcSi137*; *egl-1*(*bc449*) animals have on average 2.7 and 2.8 extra cells, respectively. (*bcSi137* animals have 0.07 extra cells.) (Fig. 2A). This indicates that EGL-1 activity level is less than wild type, but still sufficient for the majority of cell deaths. Using a microfluidic system and long-term cell fate tracking as described below, we also found that 26% of QL.pp inappropriately survive in *bcSi137*; *egl-1*(*bc449*) animals (Fig. 2B). Furthermore, we found that the average survival time of QL.pp that die is 238.8 min compared to 141.8 min in wild type, indicating that the apoptotic cell death process is slowed in *bcSi137*; *egl-1*(*bc449*) animals (Fig. 2C). Hence, the fusion of 18 copies of the SunTag peptide, which (including spacers) amounts to 427 amino acids, to the EGL-1 protein induces a weak *egl-1* loss-of-function phenotype. Finally, to determine whether *bcSi137*; *egl-1*(*bc449*) animals exhibit additional defects in the QL.p lineage, we measured the cell cycle length of QL.p, its ability to divide asymmetrically by size and its ability to unequally segregate mitochondria, a phenomenon that we recently described [34]. We found no defects in QL.p’s ability to divide asymmetrically by size or to unequally segregate mitochondria; however, QL.p’s average cell cycle length was increased from 128.3 min to 143.1 min (Fig. S5A–C).

To image the QL.p neuroblast lineage in *bcSi137*; *egl-1*(*bc449*) animals in real time, L1 larvae were cultured and immobilized for long-term imaging and cell fate tracking using a microfluidic device as recently described [34]. Using a Q lineage-specific reporter (*bcIs158*) and an upright Zeiss LSM 980 microscope equipped with a GaAsP detector, the QL neuroblast was identified, tracked and imaged at different time points between its division and the fate commitment of its daughter cells using the Multiplex Airyscan CO-8Y mode. (Cell divisions were identified in CO-8Y mode. Upon identification of a dividing QL.p neuroblast, the imaging mode was switched to Airyscan Super Resolution concurrently with the activation of the microfluidic immobilization system to capture the division itself. Airyscan CO-8Y mode offers greater sensitivity than LSM mode, which enables faster scanning and reduced phototoxicity. The resolving capabilities of Airyscan SR were not necessary here.) Similarly, we tracked and imaged QL.pp from its birth until it adopted morphological changes characteristic of apoptotic cells and died ( ~ 240 min post-QL.p division in *bcSi137*; *egl-1*(*bc449*) animals). Airyscan super resolution images were acquired for the time points ‘QL.p metaphase’ and ‘QL.p post-cytokinesis’ as well as the time point at which EGL-1 was first detected in CO-8Y mode (‘EGL-1 appearance’). Using this approach, we were unable to detect any discernible mScarlet signal indicative of 18xSunTag::EGL-1 protein in the cytoplasm of the neuroblast QL.p or its surviving daughter cell QL.pa in *bcSi137*; *egl-1*(*bc449*) animals (*n* = 43) (Fig. S6A); however, we were able to detect mScarlet signal indicative of 18xSunTag::EGL-1 protein in the cytoplasm of all QL.pp cells analyzed (*n* = 43) (Fig. 6B; Fig. S6A). As a control, no mScarlet signal was detected in the cytoplasm of QL.pp in *bcIs137* animals alone (Fig. S6B). Furthermore, super resolution imaging revealed that from the time it was first detected in QL.pp (‘EGL-1 appearance’), cytoplasmic mScarlet and, hence, EGL-1 protein co-localized with mitochondria ( + 76 min time point in the example shown in Fig. S6A and Fig. 6B, C, which depict the same QL.pp cell) (Fig. 6A, C). Using image processing in Fiji and 3D rendering and surface analysis algorithms in Imaris, we determined the proportion of EGL-1 protein associated with mitochondria at the ‘EGL-1 appearance’ time point in all 43 QL.pp cells analyzed and found that this amounts to 90% (Fig. 6D). Similarly, we found that 78% of EGL-1 protein spots are associated with mitochondria (Fig. 6E) and that the average volume of EGL-1 spots associated with mitochondria is significantly larger than that of EGL-1 spots not associated with mitochondria (0.24 μm^3^ versus 0.05 μm^3^) (Fig. 6F). Finally, we found that the time before EGL-1 appearance, but not the amount of EGL-1 that appeared, correlates with QL.pp survival time (Fig. 6G, H). In other words, the earlier EGL-1 protein is detected in QL.pp, the faster QL.pp dies.

Taken together, our analyses of the post-embryonic QL.p neuroblast lineage confirm that EGL-1 protein is present in the cell programmed to die but not its surviving sister and that EGL-1 protein localizes to mitochondria. They also reveal potential lineage-specific differences with respect to the presence of EGL-1 protein in mother cells and the kinetics of its appearance in the daughter programmed to die.

## Discussion

**Fig. 7:**
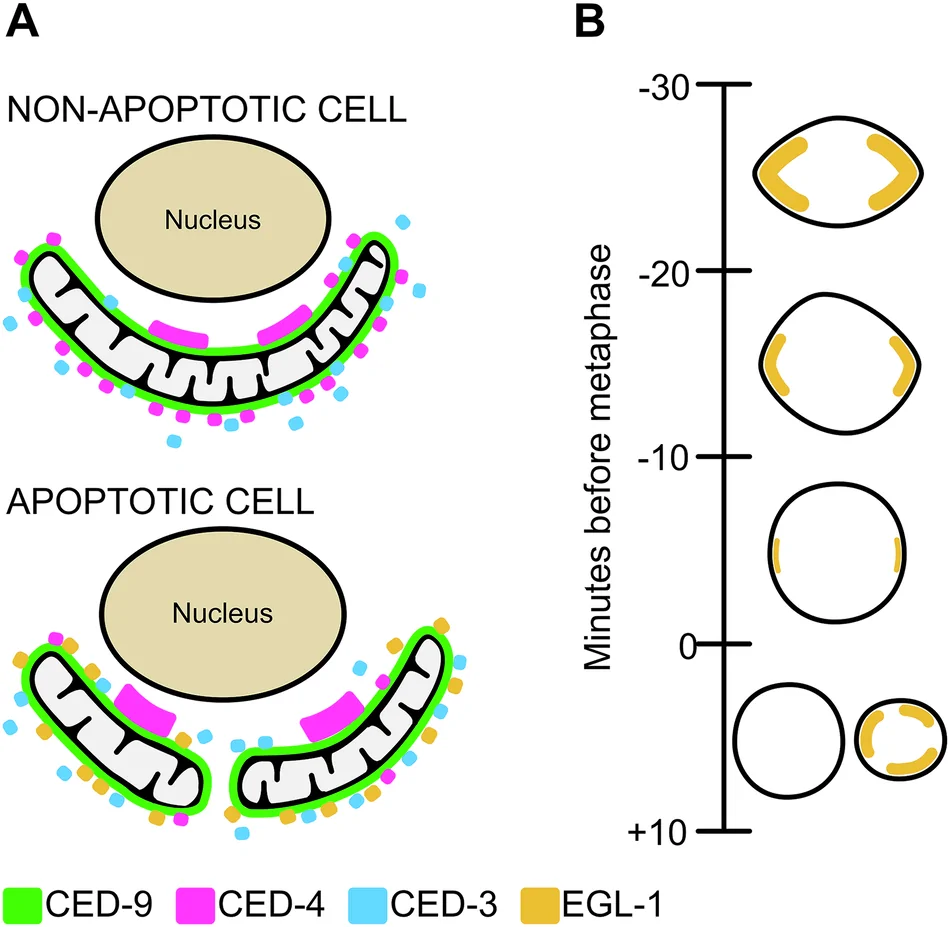
Schematic of EGL-1 BH3-only subcellular localization and levels in cell death lineages. **A** Schematic of subcellular localization of EGL-1 BH3-only, CED-9 BCL-2, CED-4 APAF-1, and CED-3 Caspase proteins in non-apoptotic and apoptotic cells in the embryo. All four proteins localize to mitochondria. **B** Schematic of dynamics of EGL-1 BH3-only levels in embryonic cell death lineages. EGL-1 protein is detectable in mothers or cells programmed to die. After their asymmetric cell division, EGL-1 protein reappears specifically in the smaller daughter cell, which is programmed to die. See text for more details.

Like its mammalian counterpart BCL-2, *C. elegans* CED-9 localizes predominantly to mitochondria [18, 19]. Mammalian BH3-only proteins associate with mitochondria in a BCL-2-dependent manner, and it has been assumed that, likewise, *C. elegans* EGL-1 associates with mitochondria in a CED-9-dependent manner; however, as pointed out in a recent review by Kim Newton and colleagues [5], the subcellular localization of EGL-1 has so far been unclear. We find that tagged versions of EGL-1 (mSG::EGL-1, 18xSunTag::EGL-1) localize predominantly to mitochondria and that EGL-1 mitochondrial localization is dependent on CED-9 (Fig. 7A). Over-expression of *egl-1* BH3-only in live embryos (which is sufficient for apoptosis) causes re-localization of the pool of CED-4 APAF-1 protein that associates with mitochondria in a CED-9 BCL-2-dependent manner into mitochondria-associated CED-4 foci [19], a step that is thought to be critical for apoptosome assembly and CED-3 Caspase activation [24, 42, 43]. The results presented here indicate that this critical step is triggered by the direct binding of EGL-1 protein to mitochondria-localized CED-9 protein (Fig. 7A).

The insertion of the mSG or 18xSunTag coding sequences could affect abundance and/or subcellular localization of the tagged EGL-1 proteins. Furthermore, the addition of mSG or 18xSunTag to EGL-1 could potentially alter the stabilities of the tagged proteins (either increase or decrease) or otherwise affect their intrinsic functions. We do not observe a major loss-of-function phenotype in the mSG-tagged strain *egl-1*(*dx243*), indicating that any such effects are either of low magnitude or have minimal impact on the function of *egl-1*. However, we do observe a weak loss-of-function phenotype in the 18xSunTag-tagged strain *egl-1*(*bc449*), which suggests that *egl-1* function is compromised.

We recently presented evidence that CED-9 BCL-2, CED-4 Apaf-1, and CED-3 Caspase proteins are ubiquitously present in *C. elegans* embryos. Here we present evidence that, in contrast, EGL-1 BH3-only protein is not ubiquitously present in *C. elegans* embryos but is found predominantly in cell death lineages, i.e., lineages in which a programmed cell death occurs. Specifically, we found that EGL-1 protein is present in cells programmed to die. We also found that EGL-1 is present in the progenitor of a cell programmed to die during embryonic development (NSM neuroblast), but we did not obtain evidence that EGL-1 is present in the progenitor of a cell programmed to die during post-embryonic development (QL.p neuroblast). Interestingly, the loss of *egl-1* compromises the ability of both of these neuroblasts to divide asymmetrically by size [44]. This suggests that a very low level of EGL-1 protein is present in QL.p as well, possibly transiently. Similarly, EGL-1 protein was detectable within 30 min after birth in the cell programmed to die during embryonic development (NSMsc) but was detected no earlier than 76 min after birth in the cell programmed to die during post-embryonic development (QL.pp). Indeed, this difference in the kinetics with which EGL-1 appears in these two cells may explain why in wild type, the embryonic NSMsc dies within ~22 min but the post-embryonic QL.pp only within ~146 min. It has previously been demonstrated that *egl-1* transcription is controlled by lineage-specific transcription factors [20, 24, 45–47]. Therefore, we propose that, rather than reflecting mechanistic differences in the execution of the apoptotic program, the differences in EGL-1 levels and kinetics observed in these two cell death lineages are the result of lineage-specific differences in *egl-1* transcription.

Our real time analysis of the NSMnb lineage using EGL-1 tagged with mSG (which is known to mature and become fluorescent within minutes [26]) suggests that the presence of EGL-1 protein in embryonic cell death lineages is highly dynamic: prior to NSMnb division, EGL-1 protein present in the NSMnb rapidly disappears; conversely, post NSMnb division, EGL-1 protein rapidly reappears in the NSMsc, which is programmed to die (Fig. 7B). Importantly, these observations hint at the existence of additional levels of the control of *egl-1* function. The combined control of *egl-1* expression at the epigenetic, transcriptional, and post-transcriptional levels results in a low concentration of *egl-1* transcripts in mothers of cells programmed to die, a higher concentration of *egl-1* transcripts in the daughter programmed to die and essentially no *egl-1* transcripts in the surviving daughter [20–22]. Based on the results presented here, we propose that *egl-1* function in mother cells is additionally controlled at the post-translational level (i.e., protein degradation), presumably to avoid inheritance of EGL-1 protein to the surviving daughter cell. Interestingly, the Anaphase Promoting Complex Cdc20 (APC^Cdc20^) has previously been shown to target the mammalian BH3-only protein Bim for ubiquitination and proteasomal degradation [48]. Therefore, cell cycle stage-dependent protein degradation might be a conserved mechanism to control the activity of BH3-only proteins. Furthermore, we propose that *egl-1* function in the daughter programmed to die is additionally controlled at the translational level (i.e., enhanced synthesis), possibly to ensure the swift removal of the cell through apoptosis. The mechanisms involved in EGL-1 degradation and enhanced EGL-1 translation remain to be explored and provide exciting opportunities for future research; indeed, our real time analysis of EGL-1 in the post-embryonic QL.p lineage using the SunTag system (which enables visualization of EGL-1 protein immediately upon translation [38–41]) hints at the possibility of localized EGL-1 translation at mitochondria.

Studies of apoptosis in *C. elegans*, a model exceptionally well suited for analyses of this conserved process, continue to uncover novel aspects of apoptosis, including how this process is activated in vivo.

## Methods

### *C. elegans* culture

Standard methods were used for *C. elegans* culture [49]. As a food source, we used the bacterial strains OP50 or AMA1004 [50]. Unless noted otherwise, all *C. elegans* strains were cultured at 20 °C.

### *C. elegans* mutations, transgenes and strains used

Bristol N2 was used as the wild-type strain, and standard methods were used for genetic manipulations [49]. Throughout our work, we used information and tools available on WormBase (https://wormbase.org/#012-34-5) [51, 52].

#### Mutations and transgenes used in this study are

**LGII:** *bcSi137* (*egl-17*p::scFv::mScarlet::GB1::NLS::*pie-1* 3′ UTR) (this study).

**LGIII:** *ced-9*(*dx210*) [19], *ced-9*(*n6731*) [31], *rdvIs1* (*egl-17*p::Myri-mCherry::*pie-1* 3’UTR + *egl-17*p::*mig-10*::YFP::*unc-54* 3’UTR + *egl-17*p::mCherry-TEV-S::*his-24* + *rol-6(su1006)*) [36].

**LGIV:** *ced-3*(*dx211*) [19], *bcIs158* (*toe-2*p::mtGFP::*unc-54* 3’UTR + *egl-17*p::Myri-SFmTurquoise2ox::*pie-1* 3’UTR + *egl-17*p::SFmTurquoise2ox-TEV-S::*his-24* + *rol-6*(*su1006*)) [34].

**LGV:** *egl-1*(*n1084n3082*) [14], *egl-1(dx243)* (this study), *egl-1(bc449)* (this study), *ltIs44* (*pie-1*p::mCherry::PH(PLC1δ1) + *unc-119*( + )) [53].

#### Strains used in this study are

MD5016*egl-1(dx243) V*

MT8735*egl-1(n1084n3082) V*

MD5034*ced-9(n6731) III; egl-1(dx243) V*

EJ1468*unc-29(e193) I; ced-9(dx212) III; ced-3(dx213) IV; egl-1(dx243) V*

MD5038*unc-29(e193) I; ced-3(dx213) IV; egl-1(dx243) V*

MD5027*ltIs44 egl-1(dx243) V*

MD4992*rdvIs1 III; egl-1(dx243) V*

MD4859*egl-1(bc449) V*

MD4919*bcSi137 II*

MD4933*bcSi137 II; bcIs158*

MD4934*bcSi137 II; egl-1(bc449) V; bcIs158*

### Plasmid constructions

To generate the plasmid **pBC1988** (hsp-16.41p::scFv::sfGFP::GB1::NLS::*unc-54* 3′ UTR), a 1.8 kb scFv::sfGFP::GB1::NLS DNA fragment was amplified by PCR from plasmid PL234 (provided by the Mounia Lagha lab) [38] using primers oYJ86 and oYJ87 (Table S1). This fragment was inserted in the backbone plasmid pPD49.83, which contains the heatshock-inducible promoter *hsp-16.41* (Fire Lab *C. elegans* Vector Kit 1995, Addgene plasmid #1448), at the SmaI site using the NEBuilder HiFi DNA Assembly Master Mix (NEB, #E2621L).

To generate plasmid **pBC1990** (*hsp-16.41*p::scFv::sfGFP::GB1::NLS::*unc-54* 3′ UTR), a 3 kb *hsp-16.41*p::scFv::sfGFP::GB1::NLS::*unc-54* 3′ UTR DNA fragment was amplified by PCR from pBC1988 using the primers oYJ105 and oYJ106 (Table S1). This fragment was assembled into the MosSCI backbone plasmid pCFJ350 (Addgene plasmid #34866) [54] between BsiWI and AvrII sites using the Hi-T4 DNA ligase (NEB, # M2622S).

To generate plasmid **pBC2000** (*hsp-16.41*p::scFv::sfGFP::GB1::NLS::*unc-54* 3′ UTR), a 1.8 kb scFv::sfGFP::GB1::NLS DNA fragment was modified by codon optimization using the *C. elegans* codon adaptor (worm.mpi-cbg.de/codons/cgi-bin/optimize.py) [55] and synthesized using the Geneart Service (Sigma). This codon-optimized scFv::sfGFP::GB1::NLS fragment was inserted into pBC1990 between PspOMI and NheI sites using the NEBuilder HiFi DNA Assembly Master Mix (NEB, #E2621L). Through this, the old version of scFv::sfGFP::GB1::NLS fragment was replaced by the codon optimized one.

To construct plasmid **pBC2005** (*hsp-16.41*p::*egl-1*::*egl-1* 3′ UTR), the 1.3 kb *egl-1* transcription unit including the UTRs was amplified by PCR from pBC08A (generated by Barbara Conradt) using primers oYJ149 and oYJ150 (Table S1). The backbone plasmid pPD49.83 that contains the heatshock-inducible promoter hsp-16.41 (Fire Lab *C. elegans* Vector Kit 1995, Addgene plasmid #1448) was linearized by PCR using primers oYJ151 and oYJ152 (Table S1). The two fragments were assembled using the NEBuilder HiFi DNA Assembly Master Mix (NEB, #E2621L).

To generate the plasmid **pBC2006** (*hsp-16.41*p::24xSunTag::*egl-1*::*egl-1* 3′ UTR), a 1.8 kb 24xSunTag DNA fragment was amplified by PCR from the SunTag-containing plasmid provided by the Suzan Ruijtenberg lab [40] using primers oYJ159 and oYJ160 (Table S1), and the plasmid pBC2005 was linearized by PCR using primers oYJ165 and oYJ166 (Table S1). The two fragments were assembled using the NEBuilder HiFi DNA Assembly Master Mix (NEB, #E2621L).

To generate the plasmid **pBC2017** (*egl-17p*::scFv::sfGFP::GBI::NLS::*pie-1* 3’UTR), a 2 kb scFv::sfGFP::GB1::NLS DNA fragment was amplified by PCR from pBC2000 (*hsp16.41*p::scFv::sfGFP::GB1::NLS::*unc-54* 3’UTR) using the primers oYJ178 and oYJ179 (Table S1). The plasmid pBC1974 [35] was linearized by PCR using the primers IS *pie-1*-3UTR-F and P*egl-17*-R (Table S1). The two fragments were assembled using the NEBuilder HiFi DNA Assembly Master Mix (NEB, #E2621L).

To generate plasmid **pBC2020** (*egl-17p*::scFv::mScarlet::GB1::NLS::*pie-1* 3′ UTR), a 5.5 kb *egl-17*p::scFv DNA fragment was amplified by PCR from pBC2017 using the primers oYJ182 and oYJ183, a 798 bp mScarlet fragment was amplified by PCR from pBC2010 (regionF1::*pes-10*p::SV40NLS::mScarlet::EGL-13NLS::*unc-53* 3’UTR) using the primers oYJ184 and oYJ185, and a 403 bp GB1::NLS::*pie-1* 3′ UTR fragment was amplified by PCR from pBC2017 using the primers oYJ186 and oYJ187 (Table S1). The MosSCI backbone plasmid pCFJ350 (Addgene plasmid #34866) [54] was linearized using restriction enzymes BsiWI and AvrII. All these fragments were assembled using the NEBuilder HiFi DNA Assembly Master Mix (NEB, #E2621L).

### CRISPR-Cas-mediated editing of the endogenous *egl-1* locus

#### CRISPR-Cas12-mediated generation of the *dx243* allele

Codon optimization and intron placement for mStayGold were determined using the *C. elegans* codon adapter tool (https://worm.mpi-cbg.de/codons/cgi-bin/optimize.py) [55]. The amino acid sequence of EGL-1(*dx243*) at the insertion site is MLMLT**MASTP……LEAHLGSAGGTGSG**LTFASTS, where bold sequences indicate the mStayGold insertion. Oligonucleotides for PCR were from Merck Life Science (UK). Q5 and LongAmp DNA polymerase were from NEB (UK). CRISPR editing was done essentially as described by Paix et al. [56], but ssDNA repair template (generated by asymmetric PCR using oligonucleotides L829 and L830) was used based on the recommendation of Eroglu et al. [57]. Cas12a and TracrRNA for use in CRISPR editing were obtained from IDT (Coralville, Iowa). Cas12a crRNA sequences were identified using CRISPOR (http://crispor.gi.ucsc.edu/crispor.py) [58].

#### CRISPR-Cas12-mediated generation of the *bc449* allele

The SunTag repeats [40] were inserted into the endogenous *egl-1* gene at the N-terminus after the 3rd start codon using CRISPR-Cas12-mediated technology [59] generating the allele *bc449*. The guide sequence of the sgRNA used was 5′-CCTCAACCTCTTCGGATCTTCTA-3′. A 1.8 kb single-strand DNA (ssDNA) repair template, which carries 24xSunTag repeats flanked by 48 nt *egl-1* homology sequence, was prepared using asymmetric PCR with the single primer oYJ163 (Table S1). The dsDNA template used for the asymmetric PCR was amplified from plasmid pBC2006 (*hsp-16.41*p::24xSunTag::*egl-1*::*egl-1* 3′ UTR; see above plasmid construction) using primers oYJ159 and oYJ160 (Table S1). Microinjections of Cas12a-sgRNA ribonucleoproteins with the ssDNA repair template into the germline and screening for successful genome editing were performed as previously described [60]. 18x SunTag repeats were successfully inserted into the *egl-1* locus. The open reading frame (ORF) was verified by Sanger sequencing.

### MosSCI-mediated generation of the *bcSi137* allele

To generate a single-copy insertion of the transgene *egl-17*p::scFv::mScarlet::GB1::NLS::*pie-1* 3′ UTR (from pBC2020), the universal MosSCI strain EG8079 [oxTi179 II; *unc-119*(ed3) III] was used for germline microinjection and targeted insertion of the transgene onto chromosome II [61]. pBC2020 was injected at 40 ng/μl with 50 ng/μl pCFJ601 (*eft-3*pMos1 transposase) (Addgene plasmid #34874) [61] and 2.5 ng/μl pPD118.33 (*myo-*2p::GFP) (Fire Lab *C. elegans* Vector Kit 1997; Addgene plasmid #1596). The genotype of the allele generated, *bcSi137*, was verified phenotypically and by PCR.

### Cell death assays

The number of extra cells in the anterior pharynx of L4 stage animals was determined using DIC microscopy as described [27]. Animals of a given genotype were randomly picked for analysis. QL.pp cell death was determined by counting the number of QL.pp disappearing or assuming a morphology characteristic of apoptotic cells [34] at the end of each microfluidics recording. Animals of a given genotype were randomly selected and QL.p lineages recorded in Multiplex Airyscan mode (see below) for at least 10 h (600 min) after the last QL.p division was recorded. This ensured that all cell death events were captured, even those that were significantly delayed (approx. 480 min).

### Microscopy

#### Widefield microscopy—presence of mSG::EGL-1 in live embryos (Fig. 3, S1-S3)

Widefield imaging was performed using a Zeiss Axio Imager M2 equipped with an EC Plan-Neofluar 100×/1.3 oil immersion objective. Images were acquired with a Photometrics Prime BSI sCMOS camera (USA) using ZEN Blue 2.6 software. Differential interference contrast (DIC) imaging was conducted using standard optics. For mSG imaging, a Colibri 5/7 LED light source was used at a wavelength of 457 nm and an intensity of 25%, in combination with filter set 90. For mCherry imaging, the same light source and filter set were used, with a wavelength of 555 nm and an intensity of 70%. The LED light source intensity was kept consistent across experiments, and all mSG images presented in the figures were adjusted to the same brightness scale using ImageJ. To prepare embryos for imaging, at least 10 randomly selected gravid adults were dissected in M9 buffer, releasing embryos at mixed developmental stages. Using a mouth pipette, *egl-1*(*dx243*) embryos were transferred to 2% agarose pads. A coverslip was then placed on top of the pad and sealed with petroleum jelly. The slides were incubated at 25 °C until the embryos had reached the desired developmental stage. Representative examples are shown in figures.

#### Super-resolution microscopy—colocalization of mSG::EGL-1 with TMRE and mScarlet::CED-9 in live embryos (Fig. 4)

TMRE was from Molecular Probes (Thermo Fisher Scientific). TMRE staining was done by culturing worms overnight on seeded NGM plates containing 100 nM TMRE and imaging randomly selected embryos mounted on 3% agarose pads. For super-resolution imaging, we used a Zeiss LSM 980 plus Airyscan 2 detector system. Imaging of mSG was done using 488 nm excitation and >509 nm emission. Imaging of mScarlet and TMRE were done separately from mSG using 552 nm excitation and >578 nm emission. Acquisition parameters were adjusted in ZEN Blue 3.3 as necessary to optimize signal/noise ratio without excessive photobleaching. Standard autofilter settings were used for Airyscan processing. At least ten embryos were imaged for each genotype and experimental condition reported. Representative examples are shown in figures.

#### Super-resolution microscopy—mSG::EGL-1 analysis in the embryonic NSMnb lineage in live embryos (Fig. 5, S4)

Super-resolution imaging was performed using a Zeiss LSM 980 microscope equipped with an Airyscan 2 detector. A 63×/1.4 NA oil-immersion objective lens combined with a 12× zoom was used. For imaging the NSMnb and its daughter cells (NSM and NSMsc), Z-stacks were acquired with a step size of 0.19 μm to capture the entire cell volumes. mSG imaging was conducted using an excitation wavelength of 488 nm at 1.0% intensity, while mCherry imaging used a wavelength of 594 nm at 2% intensity. Acquisition parameters in ZEN Blue 2.6 were adjusted to optimize the signal-to-noise ratio and minimize photobleaching. Airyscan processing was performed using standard autofilter settings. Randomly selected embryos were mounted following the widefield microscopy protocol described above, with a 2–3 h incubation at 25 °C to obtain comma-stage embryos. At this stage, the NSMnb can be identified using a plasma membrane marker, based on its distinct position and morphology [32]. To visualise EGL-1 protein in the NSM lineage, the CRISPR allele *dx243* (mSG::EGL-1) was used. The transgene *ltIs44* (*pie-1*p::mCherry::PH(PLC1δ1)) was employed to label cell boundaries [53]. Quantification of mSG::EGL-1 signal intensity (Fig. 5B, D) was conducted using ImageJ. A region of interest (ROI) was drawn around the cell boundary in the mCherry channel. This was repeated for each Z-slice. The mean gray value and area within each ROI was measured in the mSG channel. The total area and total mean gray values were summed across each Z-slice. The total mean gray value across the Z-slices was then divided by the total area across the Z-slices. Number of NSM lineages analyzed *n* = 13. Representative examples are shown in figures.

#### Super-resolution imaging—mSG::EGL-1 analysis in the post-embryonic QL.p lineage in live L1 larvae

Randomly selected animals were immobilized using polystyrene nanospheres, as described [34]. We used *egl-1*(*dx243)* animals carrying the transgene *rdvIs1* (*egl-17*p::Myri-mCherry::*pie-1* 3’UTR + *egl-17*p::*mig-10*::YFP::*unc-54* 3’UTR + *egl-17*p::mCherry-TEV-S::*his-24* + *rol-6(su1006)*) [36]. We used an inverted Zeiss LSM 980 with Airyscan 2 detector equipped with a GaAsP detector and a Zeiss C Plan Apochromat 63x/1.40 objective. We imaged QL.p at different cell cycle stages between QL division and QL.p division in Airyscan super resolution mode. mCherry and mSG were excited at 594 nm (laser power = 0.8%), and 488 nm (laser power = 0.8%), respectively, and the detection wavelength was 300–720 nm for both channels. We adjusted other settings as follows: FOV = 16.94 × 16.94 µm (398 × 398 pixels), voxel size = 0.043 × 0.043 × 0.130 µm, pixel time = 0.82 µs (frame time = 340.30 ms), detector gain = 850 V, and scan direction = bidirectional (no averaging). After completing the experiment, recordings were 2D Airyscan-processed (standard strength) in ZenBlue 3.3. Airyscan-processing was necessary to visualize and process images in ImageJ (image alignment, cropping, and 2D projection with maximum intensity projection). Number of animals analysed *n* = 111.

#### Super-resolution imaging and 3D image analysis—18xSunTag::EGL-1 analysis in the QL.p lineage in live L1 larvae (Fig. 6, S5, S6)

Randomly selected L1 larvae were immobilized using a microfluidics device [62], as described [34]. We used wild-type and *egl-1(bc449)* animals both homozygous for the transgenes *bcIs158* (*toe-2*p::mtGFP::*unc-54* 3’UTR^4^ + *egl-17*p::Myri-SFmTurquoise2ox::*pie-1* 3’UTR^4^ + *egl-17*p::SFmTurquoise2ox-TEV-S::*his-24*^4^ + *rol-6*(*su1006*)) [63] and *bcSi137* (*egl-17*p::scFv::mScarlet::GB1::NLS::*pie-1* 3′ UTR). We used an inverted LSM 980 equipped with an Airyscan 2 and GaAsP detector. We followed the entire QL.p lineage through Multiplex Airyscan CO-8Y (long-term imaging) imaging and acquired Airyscan super resolution images (see below) for the time points ‘QL.p metaphase’, ‘QL.p post-cytokinesis’, and ‘EGL-1 appearance’ (only in *egl-1(bc449)* animals). We performed Multiplex Airyscan with a Zeiss C Plan Apochromat 63x/1.40 objective using the Q lineage marker (*bcIs158*, SFmTurquoise2ox) and scFv::mScarlet::NLS (*bcSi137*). SFmTurquoise2ox and mScarlet were excited at 445 nm (laser power at 1.2%, which is equal to 0.12% in standard LSM mode) and 561 nm (laser power at 1.5%, which is equal to 0.15% in standard LSM mode), respectively. The detection wavelengths were 462–720 nm. The tile function was used to save multiple XYZ positions so that positions (P1, P2, P3, …) were organized and assigned across the microfluidic device geometry. We adjusted other settings as follows: FOV = 133.46 × 34.57 µm (1992 × 516 pixels), voxel size = 0.078 × 0.078 × 0.500 µm, pixel time = 1.53 µs (frame time = 206.23 ms), detector gain = 990 V, and scan direction = bidirectional (no averaging). After completing the experiment, recordings were 2D Airyscan-processed (standard strength) in ZenBlue 3.3. Airyscan-processing was necessary to visualize and process images in ImageJ. Super-resolution imaging was performed using the same Zeiss C Plan Apochromat 63x/1.40 objective. SFmTurquoise2ox, mScarlet and mtGFP fluorophores were excited at 405 nm (1.0%), 561 nm (1.0%), and 488 nm (1.0%), respectively, while their detection ranges were always 300–720 nm. We adjusted other settings as follows: FOV = 20.66 × 7.12 µm (464 × 160 pixels), voxel size = 0.045 × 0.045 × 0.130 µm, pixel time = 0.71 µs (frame time = 297.80 ms), detector gain = 990 V, and scan direction = bidirectional (no averaging). Z-stacks were Airyscan-processed (2D, standard strength) before export. Images were processed (SFmTurquoise2ox and mtGFP channels) in Fiji and 3D rendered in Imaris according to Segos et al. [34]. Number of animals analysed *n* = 43. EGL-1 spot (mScarlet channel) detection was performed in 3D using the Imaris Surface algorithm set with the following specifications:

#### Algorithm

Enable Region of Interest = true

Process Entire Image = false

Enable Region Growing = false

Enable Tracking = false

Enable Shortest Distance = true

#### Region of interest

Region1: XYZT from [X Y Z T] to [X Y Z T]

Source channel

Source Channel index = 1 (mScarlet channel)

Enable Smooth = true

Surface Grain Size = 0.00100 μm

Enable Eliminate Background = true

Diameter Of Largest Sphere = 0.334 μm (mScarlet)

#### Threshold

Enable automatic threshold = true

Enable Automatic Threshold = true

Active Threshold B = false

#### Filter surfaces

Number of Voxels Img = 1” above 10.0

“In contact” and “Not in contact” EGL-1 spots, relative to mitochondria, were determined using the surface-surface contact option in the Surface algorithm. Mitochondria-contacting and -not contacting EGL-1 spots were saved as new surface files for downstream quantification of average and total surface volumes.

### Statistical analysis

Statistical analyses were performed using GraphPad Prism8.

### Figures and illustrations

Figures and schematics were generated in Affinity Designer 2.6.0, and GraphPad Prism8 and Prism10.

## Supplementary information

Supplemental material

aj-checklist_Jiang et al

## Data Availability

All data and material used in this manuscript are available and can be requested from the corresponding author.
