# Supplementary material for: Tagging of C. elegans apoptosis activator EGL-1 BH3-only reveals CED-9 BCL-2-dependent mitochondrial localization and dynamic control of EGL-1 synthesis and degradation in vivo
Source: Cell Death Differ. 2026 Feb 11;33(9):1756–67. doi: 10.1038/s41418-026-01682-0 (PMC13545084; doi:10.1038/s41418-026-01682-0)
Supplement: Supplementary file 1 [file 41418_2026_1682_MOESM1_ESM.pdf]

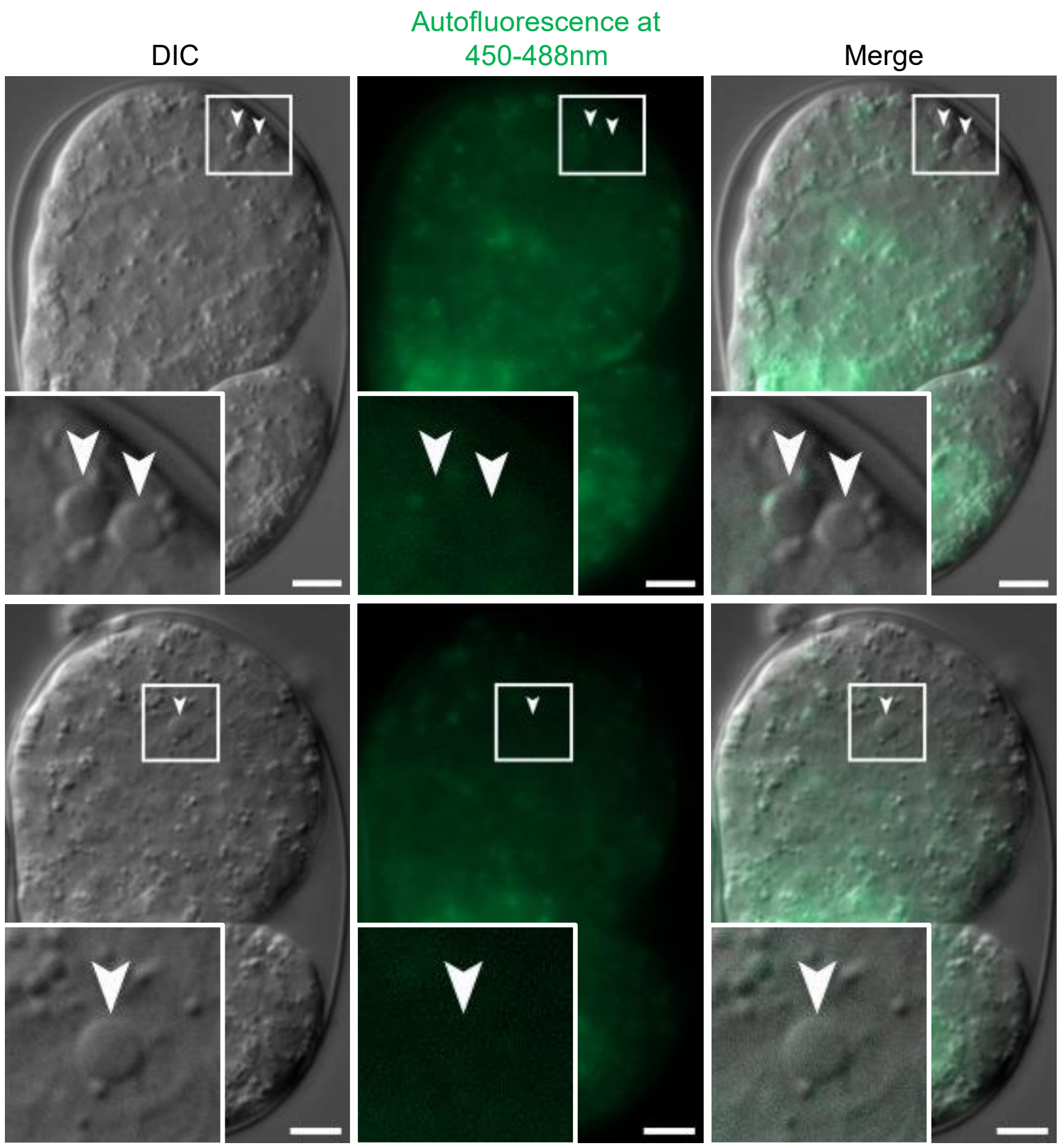

Fig S1. Autofluorescence in live control (N2) embryos.

All images were acquired on a Zeiss Axio Imager M2 microscope as described in Methods. Panels depicting green signal were set to the same brightness scale as the experimental strain containing mSG::EGL-1 (*dx243*, green) in ImageJ (see **Figure 3**). Images were colorised, composited, and annotated in ImageJ, converted to 8-bit, and compiled in Microsoft PowerPoint™. Scale bar, 2 µm. There is no green signal in cells programmed to die (cell corpses) in absence of mSG::EGL-1 (*dx243*, green). White arrows mark the location of refractile apoptotic bodies, identified using Differential Interference Contrast (DIC) microscopy.

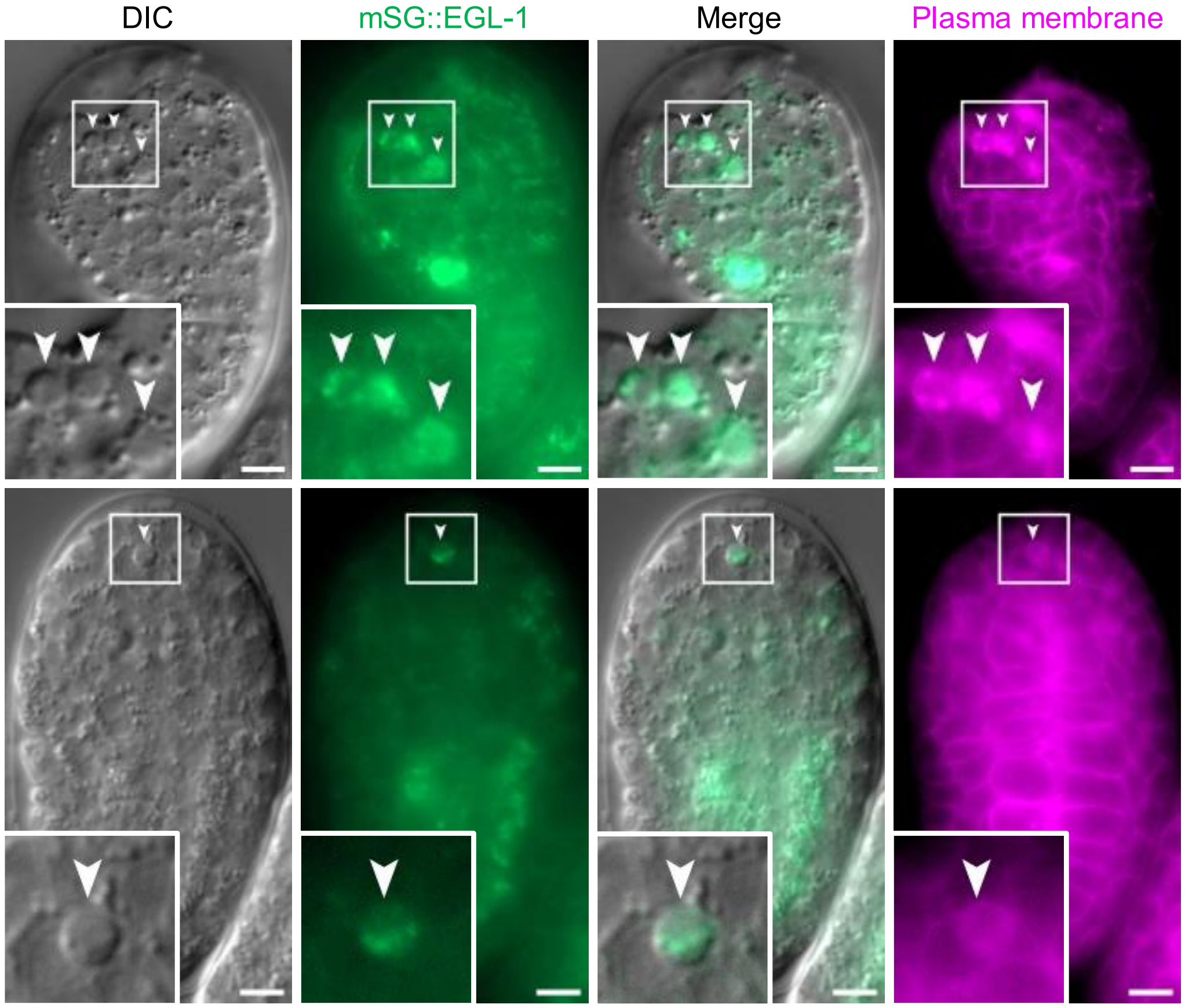

Fig S2. Presence of mSG-tagged EGL-1 in apoptotic cells in live embryos.

All images were acquired on a Zeiss Axio Imager M2 microscope as described in Methods. mSG::EGL-1 (*dx243*, green) panels were set to the same brightness scale in ImageJ. Plasma membranes were labelled with *pie-1p::mCherry::PH*(*PLC1δ1*) (*ltIs44*, magenta). Images were colorised, composited, and annotated in ImageJ, converted to 8-bit, and compiled in Microsoft PowerPoint™. White arrows mark the location of refractile apoptotic bodies, identified using Differential Interference Contrast (DIC) microscopy. Scale bar, 2 μm. Genotype of animals analysed *ltIs44 egl-1(dx243)* V.

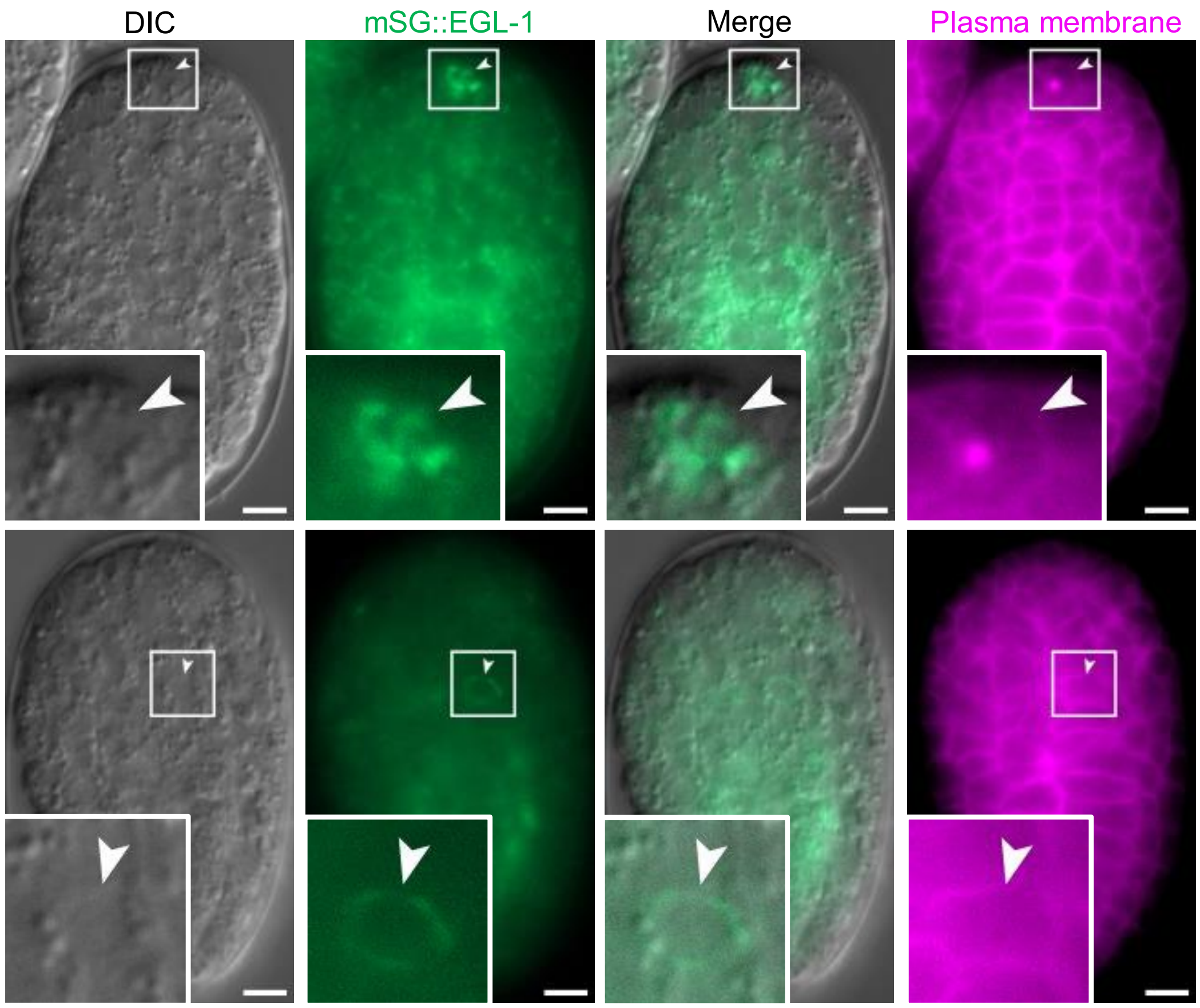

Fig S3. Presence of mSG-tagged EGL-1 in non-apoptotic cells in live embryos.

All images were acquired on a Zeiss Axio Imager M2 microscope as described in Methods. mSG::EGL-1 (*dx243*, green) panels were set to the same brightness scale in ImageJ. Plasma membranes were labelled with *pie-1p::mCherry::PH*(*PLC1δ1*) (*ltIs44*, magenta). Images were colorised, composited, and annotated in ImageJ, converted to 8-bit, and compiled in Microsoft PowerPoint™. The white arrow indicates the non-corpse cell containing mSG::EGL-1 signal, presumed to be the mother of a cell fated to die. Scale bar, 2 μm. Genotype of animals analysed *ltIs44 egl-1(dx243)* V.

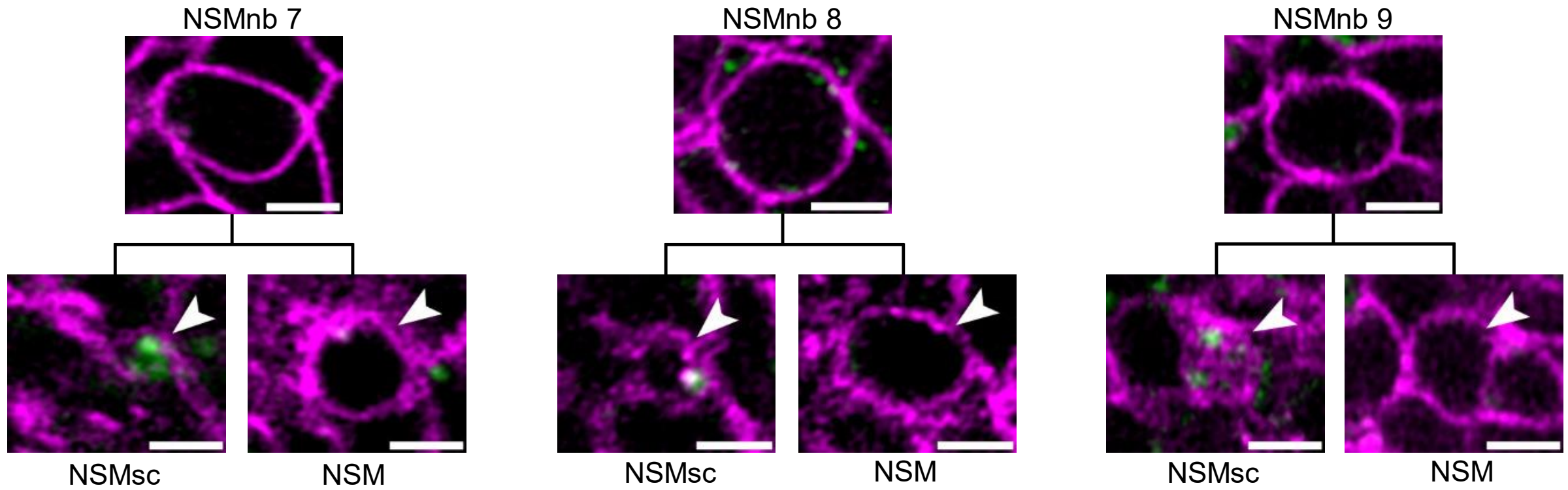

Fig S4. **mSG::EGL-1 is absent from the NSMnb at metaphase and reappears in the daughter programmed to die (NSMsc).**

Central Z-slices of representative wild-type NSMnbs at metaphase and of their daughter cells (NSMsc and NSM), expressing mSG::EGL-1 (*dx243*, cyan) and *pie-1p::mCherry::PH*(*PLC1δ1*) (*ltIs44*, magenta). Images connected by black lines derive from the same lineage. White arrows indicate the locations of the NSMsc and NSM within the image frame. Scale bar, 2 µm.

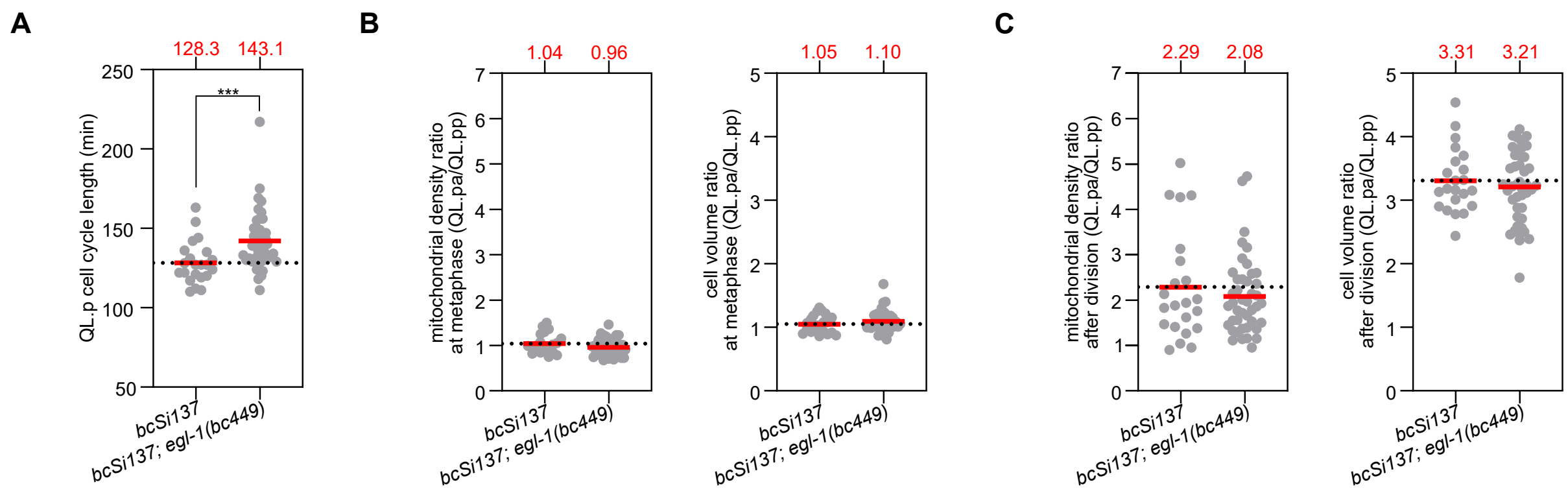

Fig S5. Effects of 18xSunTag::EGL-1 on QL.p cell cycle length and cell volume ratios (A/P) and on mitochondrial density ratios (A/P) at metaphase and post-cytokinesis.

(A) QL.p cell cycle length, (B) Mitochondrial density ratio at metaphase (QL.p A side/P side – left panel) and cell volume ratio at metaphase (QL.p A side/P side – right panel), (C) Mitochondrial density ratio after division (QL.pa/QL.pp) and cell volume ratio after division (QL.pa/QL.pp). For all plots, n=22 and n=43 for *bcSi137* and *bcSi137; egl-1(bc449)*, respectively. (Both strains also carried the transgene *bcIs158.*) P values are calculated using the paired T-test with Welch's correction (A, B left, C right) and the Mann-Whitney test (B right, C left). Normality was tested with the Shapiro-Wilk test. *: P value ≤ 0.05; **: P value ≤ 0.01; ***: P value ≤ 0.001; ****: P value ≤ 0.0001. Red bar and values indicate the population average.

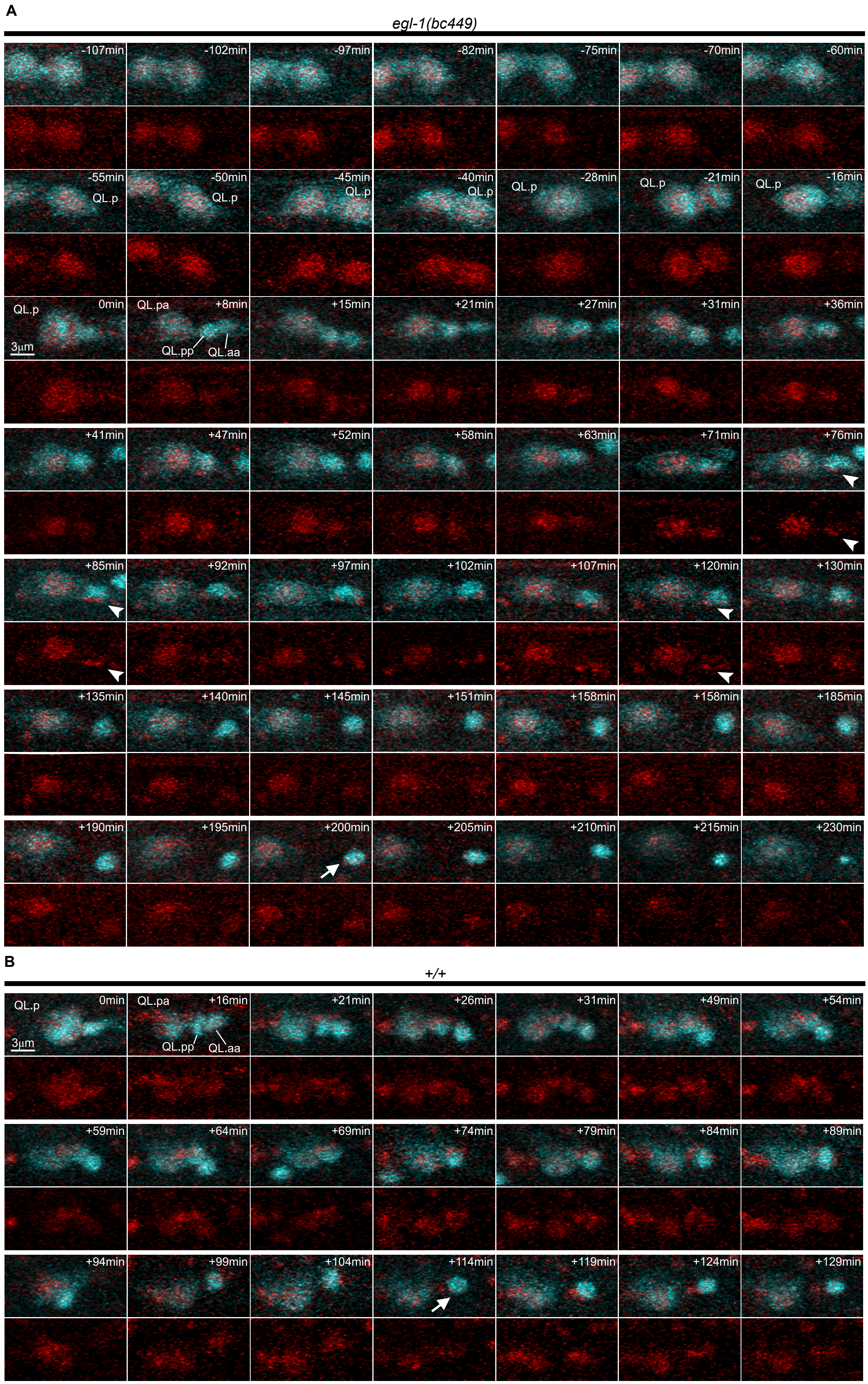
A
egl-1(bc449)
-107min
-102min
-97min
-82min
-75min
-70min
-60min
-55min
QL.p
-50min
QL.p
-45min
QL.p
-40min
QL.p
QL.p
-28min
QL.p
-21min
QL.p
-16min
QL.p
0min
3μm
QL.pa
+8min
QL.pp
QL.aa
+15min
+21min
+27min
+31min
+36min
+41min
+47min
+52min
+58min
+63min
+71min
+76min
+85min
+92min
+97min
+102min
+107min
+120min
+130min
+135min
+140min
+145min
+151min
+158min
+158min
+185min
+190min
+195min
+200min
+205min
+210min
+215min
+230min
B
+/+
QL.p
0min
3μm
QL.pa
+16min
QL.pp
QL.aa
+21min
+26min
+31min
+49min
+54min
+59min
+64min
+69min
+74min
+79min
+84min
+89min
+94min
+99min
+104min
+114min
+119min
+124min
+129min

Fig S6. Imaging of 18xSunTag::EGL-1 appearance in the post-embryonic QL.p lineage.

(A) Airyscan multiplex dual-colour confocal recording of the QL.p lineage between QL.p birth and QL.pp death in *bcSi137 II; egl-1(bc449) V; bcIs158* animals (*egl-1*(*bc449*)). Cell membrane and chromatin are shown in cyan, scFv-mScarlet-NLS is shown in red. Arrowheads indicate the appearance of EGL-1 signal (+76 min and +85 min) and the disappearance of EGL-1 signal (+120 min) in QL.pp. The arrow (+200 min) indicates QL.pp death. (B) Airyscan multiplex dual-colour confocal recording of the QL.p lineage between QL.p birth and QL.pp death in *bcSi137 II; bcIs158* animals (+/+). No EGL-1 appearance can be observed at any time point. Cell membrane and chromatin are shown in cyan, scFv-mScarlet-NLS is shown in red. The arrow (+200 min) indicates QL.pp death.

Table S1. List of oligos and RNA molecules used for cloning and mutagenesis.

**Name**	**Sequence (5′–3′)**
IS pie-1-3UTR-F	AAGACTAGTGGATCCTAATTTTGC
Pegl-17-R	AGCTCACATTTCGGGCAC
oYJ86	AAAATCCTCATCGGGATCCCCGGGATGGGCCCCGACATCGTGATGAC
oYJ87	TGGGTCCTTTGGCCAATCCCGGGTTACACCTTGCGCTTCTTCTTG
oYJ105	TCTTCGAATGCATCGCGCGCACCGTACGCCAAAAACGGAACGTTGAGCTG
oYJ106	TGACTAGAGGGTACCAGAGCTCACCTAGGAAACAGTTATGTTTGGTATATTG
oYJ149	AAAAAACTTCGAAAATCCTCATCGGGATCCAGCTGTGCACGCACACCATTC
oYJ150	TAAAAATAGGCGTATCACGAGGCCCTTAAGAGTATACAATCGTAGGCATAC
oYJ151	CTTAAGGGCCTCGTGATACG
oYJ152	GGATCCCGATGAGGATTTTCGAAG
oYJ159	ACCTTACTAATTTCCAGATGCTCACCTTTGCCTCAACCTCTTCGGATCTTCTACCAATG<br>GAAGAACTTTTGAGCAAG
oYJ160	GTTCTTTTCGTTGTAGAAAACGGAAGATTGAACGTCAAAAACGTTGGACATTGGTAGA<br>AGCTTTTTAAGTCGGGCTACTTCATTCTCG
oYJ163	ACCTTACTAATTTCCAGATGC
oYJ165	CATTGGTAGAAGATCCGAAGAG
oYJ166	TCCAACGTTTTTGACGTTCAATC
oYJ178	ATGATTTTTCAGGTGCCCGAAATGTGAGCTATGGGACCAGACATCGTCATGAC
oYJ179	AATACGGCAAAATTAGGATCCACTAGTCTTTTAGACCTTACGCTTCTTCTTTG
oYJ182	GATCTTCGAATGCATCGCGCGCACCCAGATGGATGTTTACTGCCAACTG
oYJ183	TGACGGCCTCTCCCTTGGAGACCATTCCGGATCCTCCTCCTCCTG
oYJ184	ATGGTCTCCAAGGGAGAGGCCGTC
oYJ185	CTTGTAGAGCTCGTCCATTCC
oYJ186	CGGAGGAATGGACGAGCTCTACAAGGGAGGAGGACGTACCGAGGAG
oYJ187	TTGACTAGAGGGTACCAGAGCTCACGTAAATTATTTTTGGCTTTTTTGGCAC
Cas12a guide	CCUCAACCUCUUCGGAUCUUCUA
L829	ataaaccccccttcaaaaccttactaatttccagATGCTCACCATGGCCTCCACCCCATTCAA
L830	TTGAACGTCAAAAACGTTGGACATTGGTAGAAGATCCGAAGAGGTTGAGGCgAAGGT<br>GAGgcctgatcccgtcccaccagcgctcccGAGGTGGGCCTCGAGGGTCT
Repair template for insertion of mStayGold	ataaaccccccttcaaaaccttactaatttccagATGCTCACCATGGCCTCCACCCCATTCAAGTTCCAACTCA<br>AGgtaagtttaaacatatatatactaactaaccctgattatttaaattttcagGGAACCATCAACGGAAAGTCCTTCA<br>CCGTCGAGGGAGAGGGAGAGGGAAACTCCCACGAGGGATCCCACAAGGGAAAGTACGTCTGC<br>ACCTCCGGAAAGCTCCCAATGTCCTGGGCCGCCCTCGGAACCTCCTTCGGATACGGAATGAAG<br>ACTACACCAAGTACCCATCCGGACTCAAGAACTGGTTCCACGAGGTCATGCCAGAGGGATTCAC
